# Supplementary material for: Acetyl-CoA synthetase activity is enzymatically regulated by lysine acetylation using acetyl-CoA or acetyl-phosphate as donor molecule
Source: Nat Commun. 2024 Jul 17;15:6002. doi: 10.1038/s41467-024-49952-0 (PMC11255334; doi:10.1038/s41467-024-49952-0)
Supplement: Supplementary file 1 — Supplementary Information [file 41467_2024_49952_MOESM1_ESM.pdf]

SUPPLEMENTARY INFORMATION

Acetyl-CoA synthetase activity is enzymatically regulated by lysine acetylation using acetyl -CoA or acetyl-phosphate as donor molecule

Chuan Qin[1], Leonie G. Graf[1], Kilian Striska[1], Markus Janetzky[1], Norman Geist[2], Robin Specht[3], Sabrina Schulze[1], Gottfried J. Palm[1], Britta Girbardt[1], Babett Dörre[1], Leona Berndt[1], Stefan Kemnitz[4], Mark Doerr[3], Uwe T. Bornscheuer[3], Mihaela Delcea[2], and Michael Lammers[1*]

[1]University of Greifswald, Institute of Biochemistry, Department of Synthetic and Structural Biochemistry, 17489 Greifswald, Germany
[2]University of Greifswald, Institute of Biochemistry, Department of Biophysical Chemistry, 17489 Greifswald, Germany
[3]University of Greifswald, Institute of Biochemistry, Department of Biotechnology & Enzyme Catalysis, 17489 Greifswald, Germany
[4]University of Greifswald, University Computing Center, Department for High Performance Computing, 17489 Greifswald, Germany

[*]correspondence should be addressed to Michael Lammers; Email: michael.lammers@uni-greifswald.de; Tel.: 03834-420-4356; Fax: 03834-420-4373

Supplementary Figures 1-20
Supplementary Tables 1-3

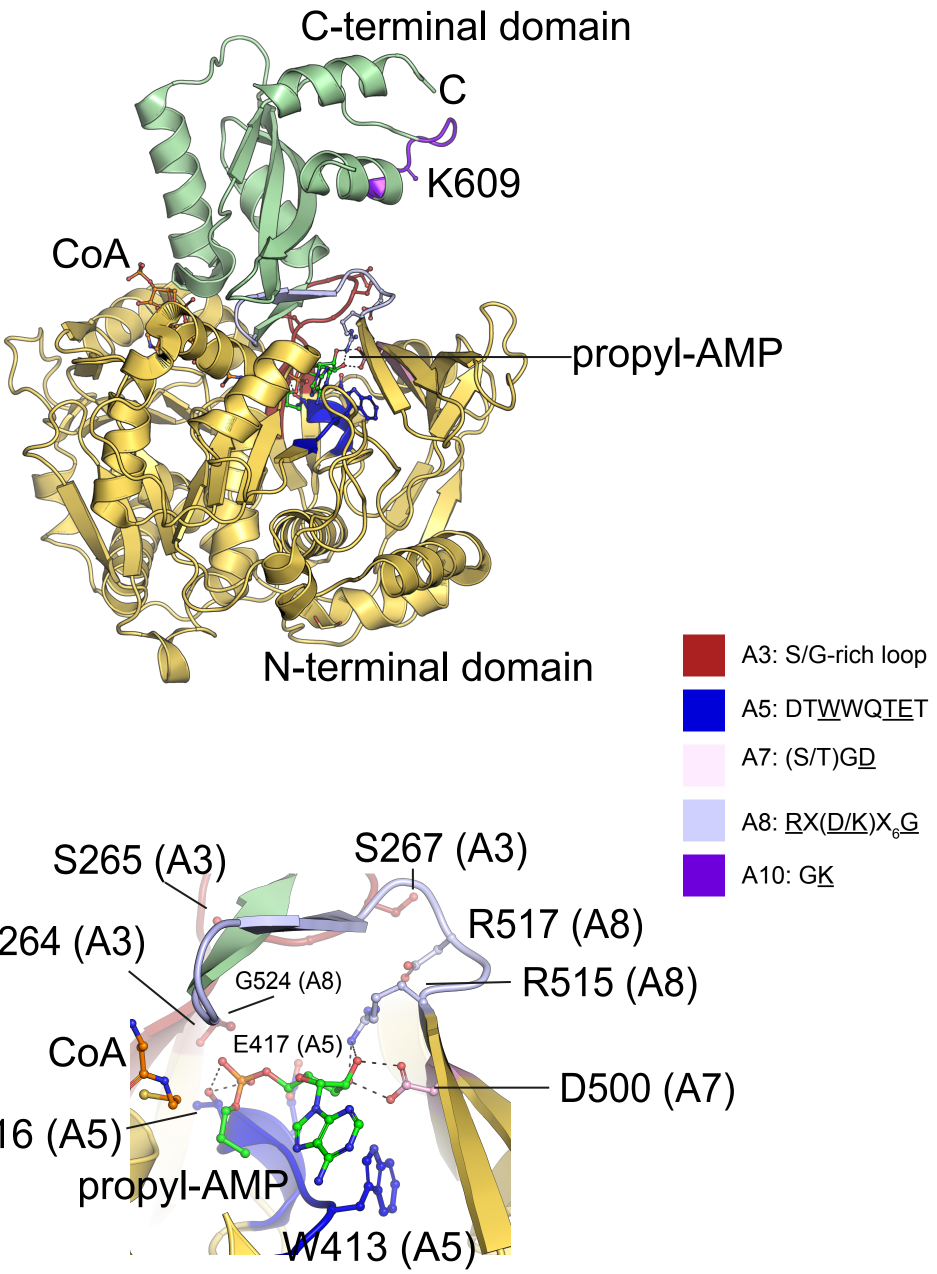

Supplementary Figure 1: Overview of conserved sequence motifs in AMP-forming acetyl-CoA synthetases exemplified on the structure of *S. enterica* AcsA in complex with CoA and propyl-AMP in the thioester-forming conformation (PDB: 1PG4 [https://doi.org/10.2210/pdb1PG4/pdb]). The core sequences A1-A10/motifs I,II,III contain important residues for catalysis as well as CoA and nucleotide binding. Core region A3/motif III: Ser-/Gly-rich loop (shown: T264, S265, S267), the phosphate binding loop important to orient the β,γ-phosphates of ATP and to contact the pyrophosphate leaving group; core region A5/motif II: a sequence containing a Thr-Glu dipeptide sequence (DTWWQTET), in which the first Trp (W413) side chain stacks to the adenine ring of ATP, Thr (T416) contacts the phosphate of acyl-AMP and the Glu (E417) coordinates a Mg^{2+} (not visible here); core region A7/motif III: has consensus sequence (S/T)GD, in which the conserved Asp (D500) binds the hydroxyls of the ATP-ribose; core region A8: conserved RX(D/K)X_6G sequence, in which the Arg (R515) contacts the hydroxyls of the acyl-AMP-ribose, Asp/Lys (D517) forms a hinge, Gly (G524) lines the CoA 4-phosphopantetheine tunnel in the thioester-forming conformation; core region A10: the region containing a conserved Gly-Lys dipeptide (K609), i.e. the lysine being acetylated located in the active site in the adenylation-conformation (Supplementary Fig. 7,12,13).

a

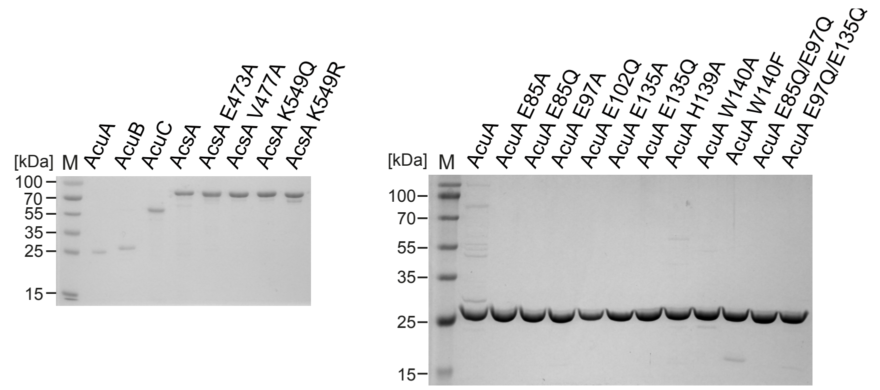

b

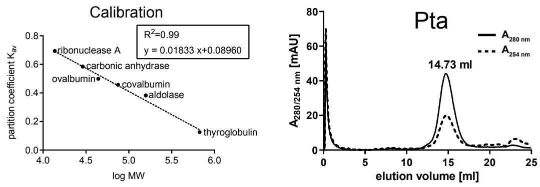

c

protein	exp. MW [Da]	elution volume [mL]	calc. MW [Da]	oligomeric state [calc. MW/exp. MW]
AcuA	25484	17.68	23665	0.93
AcsA	66043	13.75	147678	2.24
Pta	35202	14.73	93544	2.66
AcsA•AcuA	91527	12.94	215385	2.35
AcsA•AcuA (CoA)	91527	13.20	190812	2.08
AcsA•AcuA (AcP)	91527	12.94	215385	2.35

d

complex	interface area [Å²]	buried area upon complex formation [Å²]
AcsA•AcsA	789.7	1579.4
AcsA•AcuA	1764.6	3529.2
AcsA K549Q•AcuA	1505.8	3011.6
AcsA K549R•AcuA	1771.5	3542.9

e

dissociated complexes	protein	exp. MW [Da]	elution volume [mL]	calc. MW [Da]	oligomeric state [calc. MW/exp. MW]
AcsA•AcuA (acetyl-CoA)	AcsA	66043	13.63	156170	2.36
	AcuA	25484	17.48	25976	1.02
AcsA•AcuA (AcP+CoA)	AcsA	66043	13.50	165921	2.51
	AcuA	25484	17.41	26837	1.04

f

protein/complex	pLDDT	model confidence (ipTM+pTM)
AcuA	94.8	
AcsA	92.1	
AcsA K549Q	92.7	
AcsA K549R	92.2	
AcsA•AcsA		0.91
AcsA•AcuA		0.72
AcsA E473A•AcuA		0.81
AcsA V477A•AcuA		0.74
AcsA K549Q•AcuA		0.45
AcsA K549R•AcuA		0.77
AcsA•AcuA E85A		0.77
AcsA•AcuA E85Q		0.78
AcsA•AcuA E97A		0.79
AcsA•AcuA E102Q		0.74
AcsA•AcuA E135A		0.61
AcsA•AcuA E135Q		0.68
AcsA•AcuA H139A		0.81
AcsA•AcuA W140A		0.71
AcsA•AcuA W140F		0.77
AcsA•AcuA E85QE97Q		0.72
AcsA•AcuA E97QE135Q		0.72

g

protein/complex	r.m.s.d. [Å]
AcsA from AcsA•AcsA to AcsA from AcsA•AcuA	0.323
AcsA and AcsA K549Q	0.180
AcsA and AcsA K549R	0.180
AcsA•AcuA and AcsA E473A•AcuA	4.399
AcsA•AcuA and AcsA V477A•AcuA	1.890
AcsA•AcuA and AcsA K549Q•AcuA	4.719
AcsA•AcuA and AcsA K549R•AcuA	1.422
AcsA•AcuA and AcsA•AcuA E85A	0.149
AcsA•AcuA and AcsA•AcuA E85Q	0.149
AcsA•AcuA and AcsA•AcuA E97A	2.429
AcsA•AcuA and AcsA•AcuA E102A	1.249
AcsA•AcuA and AcsA•AcuA E102Q	2.462
AcsA•AcuA and AcsA•AcuA E97Q	2.487
AcsA•AcuA and AcsA•AcuA E135A	5.366
AcsA•AcuA and AcsA•AcuA E135Q	1.257
AcsA•AcuA and AcsA•AcuA H139A	2.801
AcsA•AcuA and AcsA•AcuA W140A	2.690
AcsA•AcuA and AcsA•AcuA W140F	2.691
AcsA•AcuA and AcsA•AcuA E85Q E97Q	4.842
AcsA•AcuA and AcsA•AcuA E97Q E135Q	2.669

Supplementary Figure 2: Tables with important numbers for calculated molecular weights, interface areas of complexes and quality scores of AlphaFold2 structure predictions.

a Final purity of the proteins AcuA, AcuB, AcuC and AcsA/AcsA mutants (left panel) and all AcuA mutants purified in this study (right panel). The lane labelled with M represents the protein molecular weight marker. Source data are provided in Supplementary Fig. 15.

b,c,e Experimental determination of the apparent molecular weights of the proteins and protein complexes studied here by analytical SEC. Source data are provided as Source Data file.

d Interface areas of the complexes.

f Confidence scores of the AlphaFold2 structure predictions (pLDDT: predicted local difference test as an assessment of the local structural accuracy for monomeric proteins; pTM: indication of the overall topological accuracy, ipTM (interface pTM score): generated by AlphaFold-Multimer, which is used together with pTM to compute model scores; pTM+ipTM for multimer model accuracy, consisting of 80% pTM (predicted template modelling score) and 20% ipTM (interface pTM score).

g Structural variation of the mutated proteins and complexes thereof compared to the wildtype counterparts.

Monomer: AcsA_*B_subtilis* UniProt: P39062
MNLKALPAIEGDHNLKNYEETYRHFDWAEAEKHFSWHETGKLNAAYEAIDRHAESFRKNKVALYYKDAKRDEKYT
FKEMKEESNRAGNVLRRYGNVEKGDRVFIFMPRSPELYFIMLGAIKIGAIAGPLFEAFMEGAVKDRLENSEAKVV
VTTPELLERIPVDKLPHLQHVFVVGGEAESGTNIINYDEAAKQESTRLDIEWMDKKDGFLLHYTSGSTGTPKGVL
HVHEAMIQQYQTGKWVLDLKEEDIYWCTADPGWVTGTVYGIFAPWLNGATNVIVGGRFSPESWYGTIEQLGVNVW
YSAPTAFRMLMGAGDEMAAKYDLTSLRHVLSVGEPLNPEVIRWGHKVFNKRIHDTWWMTETGSQLICNYPCMDIK
PGSMGKPIPGVEAAIVDNQGNELPPYRMGNLAIKKGWPSMMHTIWNNPEKYESYFMPGGWYVSGDSAYMDEEGYF
WFQGRVDDVIMTSGERVGPFEVESKLVEHPAIAEAGVIGKPDPVRGEIIKAFIALREGFEPSDKLKEEIRLFVKQ
GLAAHAAPREIEFKDKLPKTRSGKIMRRVLKAWELNLPAGDLSTMED

Dimer: AcsA_*B_subtilis* UniProt: P39062
MNLKALPAIEGDHNLKNYEETYRHFDWAEAEKHFSWHETGKLNAAYEAIDRHAESFRKNKVALYYKDAKRDEKYT
FKEMKEESNRAGNVLRRYGNVEKGDRVFIFMPRSPELYFIMLGAIKIGAIAGPLFEAFMEGAVKDRLENSEAKVV
VTTPELLERIPVDKLPHLQHVFVVGGEAESGTNIINYDEAAKQESTRLDIEWMDKKDGFLLHYTSGSTGTPKGVL
HVHEAMIQQYQTGKWVLDLKEEDIYWCTADPGWVTGTVYGIFAPWLNGATNVIVGGRFSPESWYGTIEQLGVNVW
YSAPTAFRMLMGAGDEMAAKYDLTSLRHVLSVGEPLNPEVIRWGHKVFNKRIHDTWWMTETGSQLICNYPCMDIK
PGSMGKPIPGVEAAIVDNQGNELPPYRMGNLAIKKGWPSMMHTIWNNPEKYESYFMPGGWYVSGDSAYMDEEGYF
WFQGRVDDVIMTSGERVGPFEVESKLVEHPAIAEAGVIGKPDPVRGEIIKAFIALREGFEPSDKLKEEIRLFVKQ
GLAAHAAPREIEFKDKLPKTRSGKIMRRVLKAWELNLPAGDLSTMED:
MNLKALPAIEGDHNLKNYEETYRHFDWAEAEKHFSWHETGKLNAAYEAIDRHAESFRKNKVALYYKDAKRDEKYT
FKEMKEESNRAGNVLRRYGNVEKGDRVFIFMPRSPELYFIMLGAIKIGAIAGPLFEAFMEGAVKDRLENSEAKVV
VTTPELLERIPVDKLPHLQHVFVVGGEAESGTNIINYDEAAKQESTRLDIEWMDKKDGFLLHYTSGSTGTPKGVL
HVHEAMIQQYQTGKWVLDLKEEDIYWCTADPGWVTGTVYGIFAPWLNGATNVIVGGRFSPESWYGTIEQLGVNVW
YSAPTAFRMLMGAGDEMAAKYDLTSLRHVLSVGEPLNPEVIRWGHKVFNKRIHDTWWMTETGSQLICNYPCMDIK
PGSMGKPIPGVEAAIVDNQGNELPPYRMGNLAIKKGWPSMMHTIWNNPEKYESYFMPGGWYVSGDSAYMDEEGYF
WFQGRVDDVIMTSGERVGPFEVESKLVEHPAIAEAGVIGKPDPVRGEIIKAFIALREGFEPSDKLKEEIRLFVKQ
GLAAHAAPREIEFKDKLPKTRSGKIMRRVLKAWELNLPAGDLSTMED

AcsA K549Q *B. subtilis* UniProt: P39062
MNLKALPAIEGDHNLKNYEETYRHFDWAEAEKHFSWHETGKLNAAYEAIDRHAESFRKNKVALYYKDAKRDEKYT
FKEMKEESNRAGNVLRRYGNVEKGDRVFIFMPRSPELYFIMLGAIKIGAIAGPLFEAFMEGAVKDRLENSEAKVV
VTTPELLERIPVDKLPHLQHVFVVGGEAESGTNIINYDEAAKQESTRLDIEWMDKKDGFLLHYTSGSTGTPKGVL
HVHEAMIQQYQTGKWVLDLKEEDIYWCTADPGWVTGTVYGIFAPWLNGATNVIVGGRFSPESWYGTIEQLGVNVW
YSAPTAFRMLMGAGDEMAAKYDLTSLRHVLSVGEPLNPEVIRWGHKVFNKRIHDTWWMTETGSQLICNYPCMDIK
PGSMGKPIPGVEAAIVDNQGNELPPYRMGNLAIKKGWPSMMHTIWNNPEKYESYFMPGGWYVSGDSAYMDEEGYF
WFQGRVDDVIMTSGERVGPFEVESKLVEHPAIAEAGVIGKPDPVRGEIIKAFIALREGFEPSDKLKEEIRLFVKQ
GLAAHAAPREIEFKDKLPKTRSG**Q**IMRRVLKAWELNLPAGDLSTMED

AcsA K549R *B. subtilis* UniProt: P39062
MNLKALPAIEGDHNLKNYEETYRHFDWAEAEKHFSWHETGKLNAAYEAIDRHAESFRKNKVALYYKDAKRDEKYT
FKEMKEESNRAGNVLRRYGNVEKGDRVFIFMPRSPELYFIMLGAIKIGAIAGPLFEAFMEGAVKDRLENSEAKVV
VTTPELLERIPVDKLPHLQHVFVVGGEAESGTNIINYDEAAKQESTRLDIEWMDKKDGFLLHYTSGSTGTPKGVL
HVHEAMIQQYQTGKWVLDLKEEDIYWCTADPGWVTGTVYGIFAPWLNGATNVIVGGRFSPESWYGTIEQLGVNVW
YSAPTAFRMLMGAGDEMAAKYDLTSLRHVLSVGEPLNPEVIRWGHKVFNKRIHDTWWMTETGSQLICNYPCMDIK
PGSMGKPIPGVEAAIVDNQGNELPPYRMGNLAIKKGWPSMMHTIWNNPEKYESYFMPGGWYVSGDSAYMDEEGYF
WFQGRVDDVIMTSGERVGPFEVESKLVEHPAIAEAGVIGKPDPVRGEIIKAFIALREGFEPSDKLKEEIRLFVKQ
GLAAHAAPREIEFKDKLPKTRSG**R**IMRRVLKAWELNLPAGDLSTMED

Monomer: AcuA *B. subtilis* UniProt: P39065
MEHHKTYHSANIKTATGSLLIEGPVSPEDLAGYEFHKDLTAFRPPREQHEALVDIAGLPEGRIIIARDGRTIVGY
VTYLYPDPLERWSEGNMEDLIELGAIEVAPDYRGCAVGKTLLTVSMMDEQMENYIVMTTEYYWHWDLKGMKKDVW
EYRKIMEKMMNAGGLVWFATDEPEISSHPANCLMARIGKNVSQESIEQFDRLRFYHRYMY

Complex: AcsA•AcuA *B. subtilis* UniProt: P39062, P39065
MNLKALPAIEGDHNLKNYEETYRHFDWAEAEKHFSWHETGKLNAAYEAIDRHAESFRKNKVALYYKDAKRDEKYT
FKEMKEESNRAGNVLRRYGNVEKGDRVFIFMPRSPELYFIMLGAIKIGAIAGPLFEAFMEGAVKDRLENSEAKVV
VTTPELLERIPVDKLPHLQHVFVVGGEAESGTNIINYDEAAKQESTRLDIEWMDKKDGFLLHYTSGSTGTPKGVL
HVHEAMIQQYQTGKWVLDLKEEDIYWCTADPGWVTGTVYGIFAPWLNGATNVIVGGRFSPESWYGTIEQLGVNVW
YSAPTAFRMLMGAGDEMAAKYDLTSLRHVLSVGEPLNPEVIRWGHKVFNKRIHDTWWMTETGSQLICNYPCMDIK
PGSMGKPIPGVEAAIVDNQGNELPPYRMGNLAIKKGWPSMMHTIWNNPEKYESYFMPGGWYVSGDSAYMDEEGYF
WFQGRVDDVIMTSGERVGPFEVESKLVEHPAIAEAGVIGKPDPVRGEIIKAFIALREGFEPSDKLKEEIRLFVKQ
GLAAHAAPREIEFKDKLPKTRSGKIMRRVLKAWELNLPAGDLSTMED:

MEHHKTYHSANIKTATGSLLIEGPVSPEDLAGYEFHKDLTAFRPPREQHEALVDIAGLPEGRIIIARDGRTIVGY
VTYLYPDPLERWSEGNMEDLIELGAIEVAPDYRGCAVGKTLLTVSMMDEQMENYIVMTTEYYWHWDLKGMKKDVW
EYRKIMEKMMNAGGLVWFATDEPEISSHPANCLMARIGKNVSQESIEQFDRLRFYHRYMY

Complex: AcsA•AcuA E85A *B. subtilis* UniProt: P39062, P39065
MNLKALPAIEGDHNLKNYEETYRHFDWAEAEKHFSWHETGKLNAAYEAIDRHAESFRKNKVALYYKDAKRDEKYT
FKEMKEESNRAGNVLRRYGNVEKGDRVFIFMPRSPELYFIMLGAIKIGAIAGPLFEAFMEGAVKDRLENSEAKVV
VTTPELLERIPVDKLPHLQHVFVVGGEAESGTNIINYDEAAKQESTRLDIEWMDKKDGFLLHYTSGSTGTPKGVL
HVHEAMIQQYQTGKWVLDLKEEDIYWCTADPGWVTGTVYGIFAPWLNGATNVIVGGRFSPESWYGTIEQLGVNVW
YSAPTAFRMLMGAGDEMAAKYDLTSLRHVLSVGEPLNPEVIRWGHKVFNKRIHDTWWMTETGSQLICNYPCMDIK
PGSMGKPIPGVEAAIVDNQGNELPPYRMGNLAIKKGWPSMMHTIWNNPEKYESYFMPGGWYVSGDSAYMDEEGYF
WFQGRVDDVIMTSGERVGPFEVESKLVEHPAIAEAGVIGKPDPVRGEIIKAFIALREGFEPSDKLKEEIRLFVKQ
GLAAHAAPREIEFKDKLPKTRSGKIMRRVLKAWELNLPAGDLSTMED:
MEHHKTYHSANIKTATGSLLIEGPVSPEDLAGYEFHKDLTAFRPPREQHEALVDIAGLPEGRIIIARDGRTIVGY
VTYLYPDPL**A**RWSEGNMEDLIELGAIEVAPDYRGCAVGKTLLTVSMMDEQMENYIVMTTEYYWHWDLKGMKKDVW
EYRKIMEKMMNAGGLVWFATDEPEISSHPANCLMARIGKNVSQESIEQFDRLRFYHRYMY

Complex: AcsA•AcuA E85Q *B. subtilis* UniProt: P39062, P39065
MNLKALPAIEGDHNLKNYEETYRHFDWAEAEKHFSWHETGKLNAAYEAIDRHAESFRKNKVALYYKDAKRDEKYT
FKEMKEESNRAGNVLRRYGNVEKGDRVFIFMPRSPELYFIMLGAIKIGAIAGPLFEAFMEGAVKDRLENSEAKVV
VTTPELLERIPVDKLPHLQHVFVVGGEAESGTNIINYDEAAKQESTRLDIEWMDKKDGFLLHYTSGSTGTPKGVL
HVHEAMIQQYQTGKWVLDLKEEDIYWCTADPGWVTGTVYGIFAPWLNGATNVIVGGRFSPESWYGTIEQLGVNVW
YSAPTAFRMLMGAGDEMAAKYDLTSLRHVLSVGEPLNPEVIRWGHKVFNKRIHDTWWMTETGSQLICNYPCMDIK
PGSMGKPIPGVEAAIVDNQGNELPPYRMGNLAIKKGWPSMMHTIWNNPEKYESYFMPGGWYVSGDSAYMDEEGYF
WFQGRVDDVIMTSGERVGPFEVESKLVEHPAIAEAGVIGKPDPVRGEIIKAFIALREGFEPSDKLKEEIRLFVKQ
GLAAHAAPREIEFKDKLPKTRSGKIMRRVLKAWELNLPAGDLSTMED:
MEHHKTYHSANIKTATGSLLIEGPVSPEDLAGYEFHKDLTAFRPPREQHEALVDIAGLPEGRIIIARDGRTIVGY
VTYLYPDPL**Q**RWSEGNMEDLIELGAIEVAPDYRGCAVGKTLLTVSMMDEQMENYIVMTTEYYWHWDLKGMKKDVW
EYRKIMEKMMNAGGLVWFATDEPEISSHPANCLMARIGKNVSQESIEQFDRLRFYHRYMY

Complex: AcsA•AcuA E97A *B. subtilis* UniProt: P39062, P39065
MNLKALPAIEGDHNLKNYEETYRHFDWAEAEKHFSWHETGKLNAAYEAIDRHAESFRKNKVALYYKDAKRDEKYT
FKEMKEESNRAGNVLRRYGNVEKGDRVFIFMPRSPELYFIMLGAIKIGAIAGPLFEAFMEGAVKDRLENSEAKVV
VTTPELLERIPVDKLPHLQHVFVVGGEAESGTNIINYDEAAKQESTRLDIEWMDKKDGFLLHYTSGSTGTPKGVL
HVHEAMIQQYQTGKWVLDLKEEDIYWCTADPGWVTGTVYGIFAPWLNGATNVIVGGRFSPESWYGTIEQLGVNVW
YSAPTAFRMLMGAGDEMAAKYDLTSLRHVLSVGEPLNPEVIRWGHKVFNKRIHDTWWMTETGSQLICNYPCMDIK
PGSMGKPIPGVEAAIVDNQGNELPPYRMGNLAIKKGWPSMMHTIWNNPEKYESYFMPGGWYVSGDSAYMDEEGYF
WFQGRVDDVIMTSGERVGPFEVESKLVEHPAIAEAGVIGKPDPVRGEIIKAFIALREGFEPSDKLKEEIRLFVKQ
GLAAHAAPREIEFKDKLPKTRSGKIMRRVLKAWELNLPAGDLSTMED:
MEHHKTYHSANIKTATGSLLIEGPVSPEDLAGYEFHKDLTAFRPPREQHEALVDIAGLPEGRIIIARDGRTIVGY
VTYLYPDPLERWSEGNMEDLI**A**LGAIEVAPDYRGCAVGKTLLTVSMMDEQMENYIVMTTEYYWHWDLKGMKKDVW
EYRKIMEKMMNAGGLVWFATDEPEISSHPANCLMARIGKNVSQESIEQFDRLRFYHRYMY

Complex: AcsA•AcuA E97Q *B. subtilis* UniProt: P39062, P39065
MNLKALPAIEGDHNLKNYEETYRHFDWAEAEKHFSWHETGKLNAAYEAIDRHAESFRKNKVALYYKDAKRDEKYT
FKEMKEESNRAGNVLRRYGNVEKGDRVFIFMPRSPELYFIMLGAIKIGAIAGPLFEAFMEGAVKDRLENSEAKVV
VTTPELLERIPVDKLPHLQHVFVVGGEAESGTNIINYDEAAKQESTRLDIEWMDKKDGFLLHYTSGSTGTPKGVL
HVHEAMIQQYQTGKWVLDLKEEDIYWCTADPGWVTGTVYGIFAPWLNGATNVIVGGRFSPESWYGTIEQLGVNVW
YSAPTAFRMLMGAGDEMAAKYDLTSLRHVLSVGEPLNPEVIRWGHKVFNKRIHDTWWMTETGSQLICNYPCMDIK
PGSMGKPIPGVEAAIVDNQGNELPPYRMGNLAIKKGWPSMMHTIWNNPEKYESYFMPGGWYVSGDSAYMDEEGYF
WFQGRVDDVIMTSGERVGPFEVESKLVEHPAIAEAGVIGKPDPVRGEIIKAFIALREGFEPSDKLKEEIRLFVKQ
GLAAHAAPREIEFKDKLPKTRSGKIMRRVLKAWELNLPAGDLSTMED:
MEHHKTYHSANIKTATGSLLIEGPVSPEDLAGYEFHKDLTAFRPPREQHEALVDIAGLPEGRIIIARDGRTIVGY
VTYLYPDPLERWSEGNMEDLI**Q**LGAIEVAPDYRGCAVGKTLLTVSMMDEQMENYIVMTTEYYWHWDLKGMKKDVW
EYRKIMEKMMNAGGLVWFATDEPEISSHPANCLMARIGKNVSQESIEQFDRLRFYHRYMY

Complex: AcsA•AcuA E102A *B. subtilis* UniProt: P39062, P39065
MNLKALPAIEGDHNLKNYEETYRHFDWAEAEKHFSWHETGKLNAAYEAIDRHAESFRKNKVALYYKDAKRDEKYT
FKEMKEESNRAGNVLRRYGNVEKGDRVFIFMPRSPELYFIMLGAIKIGAIAGPLFEAFMEGAVKDRLENSEAKVV
VTTPELLERIPVDKLPHLQHVFVVGGEAESGTNIINYDEAAKQESTRLDIEWMDKKDGFLLHYTSGSTGTPKGVL
HVHEAMIQQYQTGKWVLDLKEEDIYWCTADPGWVTGTVYGIFAPWLNGATNVIVGGRFSPESWYGTIEQLGVNVW
YSAPTAFRMLMGAGDEMAAKYDLTSLRHVLSVGEPLNPEVIRWGHKVFNKRIHDTWWMTETGSQLICNYPCMDIK
PGSMGKPIPGVEAAIVDNQGNELPPYRMGNLAIKKGWPSMMHTIWNNPEKYESYFMPGGWYVSGDSAYMDEEGYF

WFQGRVDDVIMTSGERVGPFEVESKLVEHPAIAEAGVIGKPDPVRGEIIKAFIALREGFEPSDKLKEEIRLFVKQ
GLAAHAAPREIEFKDKLPKTRSGKIMRRVLKAWELNLPAGDLSTMED:
MEHHKTYHSANIKTATGSLLIEGPVSPEDLAGYEFHKDLTAFRPPREQHEALVDIAGLPEGRIIIARDGRTIVGY
VTYLYPDPLERWSEGNMEDLIELGAI**A**VAPDYRGCAVGKTLLTVSMMDEQMENYIVMTTEYYWHWDLKGMKKDVW
EYRKIMEKMMNAGGLVWFATDEPEISSHPANCLMARIGKNVSQESIEQFDRLRFYHRYMY

Complex: AcsA•AcuA E102Q *B. subtilis* UniProt: P39062, P39065
MNLKALPAIEGDHNLKNYEETYRHFDWAEAEKHFSWHETGKLNAAYEAIDRHAESFRKNKVALYYKDAKRDEKYT
FKEMKEESNRAGNVLRRYGNVEKGDRVFIFMPRSPELYFIMLGAIKIGAIAGPLFEAFMEGAVKDRLENSEAKVV
VTTPELLERIPVDKLPHLQHVFVVGGEAESGTNIINYDEAAKQESTRLDIEWMDKKDGFLLHYTSGSTGTPKGVL
HVHEAMIQQYQTGKWVLDLKEEDIYWCTADPGWVTGTVYGIFAPWLNGATNVIVGGRFSPESWYGTIEQLGVNVW
YSAPTAFRMLMGAGDEMAAKYDLTSLRHVLSVGEPLNPEVIRWGHKVFNKRIHDTWWMTETGSQLICNYPCMDIK
PGSMGKPIPGVEAAIVDNQGNELPPYRMGNLAIKKGWPSMMHTIWNNPEKYESYFMPGGWYVSGDSAYMDEEGYF
WFQGRVDDVIMTSGERVGPFEVESKLVEHPAIAEAGVIGKPDPVRGEIIKAFIALREGFEPSDKLKEEIRLFVKQ
GLAAHAAPREIEFKDKLPKTRSGKIMRRVLKAWELNLPAGDLSTMED:
MEHHKTYHSANIKTATGSLLIEGPVSPEDLAGYEFHKDLTAFRPPREQHEALVDIAGLPEGRIIIARDGRTIVGY
VTYLYPDPLERWSEGNMEDLIELGAI**Q**VAPDYRGCAVGKTLLTVSMMDEQMENYIVMTTEYYWHWDLKGMKKDVW
EYRKIMEKMMNAGGLVWFATDEPEISSHPANCLMARIGKNVSQESIEQFDRLRFYHRYMY

Complex: AcsA•AcuA E135A *B. subtilis* UniProt: P39062, P39065
MNLKALPAIEGDHNLKNYEETYRHFDWAEAEKHFSWHETGKLNAAYEAIDRHAESFRKNKVALYYKDAKRDEKYT
FKEMKEESNRAGNVLRRYGNVEKGDRVFIFMPRSPELYFIMLGAIKIGAIAGPLFEAFMEGAVKDRLENSEAKVV
VTTPELLERIPVDKLPHLQHVFVVGGEAESGTNIINYDEAAKQESTRLDIEWMDKKDGFLLHYTSGSTGTPKGVL
HVHEAMIQQYQTGKWVLDLKEEDIYWCTADPGWVTGTVYGIFAPWLNGATNVIVGGRFSPESWYGTIEQLGVNVW
YSAPTAFRMLMGAGDEMAAKYDLTSLRHVLSVGEPLNPEVIRWGHKVFNKRIHDTWWMTETGSQLICNYPCMDIK
PGSMGKPIPGVEAAIVDNQGNELPPYRMGNLAIKKGWPSMMHTIWNNPEKYESYFMPGGWYVSGDSAYMDEEGYF
WFQGRVDDVIMTSGERVGPFEVESKLVEHPAIAEAGVIGKPDPVRGEIIKAFIALREGFEPSDKLKEEIRLFVKQ
GLAAHAAPREIEFKDKLPKTRSGKIMRRVLKAWELNLPAGDLSTMED:
MEHHKTYHSANIKTATGSLLIEGPVSPEDLAGYEFHKDLTAFRPPREQHEALVDIAGLPEGRIIIARDGRTIVGY
VTYLYPDPLERWSEGNMEDLIELGAIEVAPDYRGCAVGKTLLTVSMMDEQMENYIVMTT**A**YYWHWDLKGMKKDVW
EYRKIMEKMMNAGGLVWFATDEPEISSHPANCLMARIGKNVSQESIEQFDRLRFYHRYMY

Complex: AcsA•AcuA E135Q *B. subtilis* UniProt: P39062, P39065
MNLKALPAIEGDHNLKNYEETYRHFDWAEAEKHFSWHETGKLNAAYEAIDRHAESFRKNKVALYYKDAKRDEKYT
FKEMKEESNRAGNVLRRYGNVEKGDRVFIFMPRSPELYFIMLGAIKIGAIAGPLFEAFMEGAVKDRLENSEAKVV
VTTPELLERIPVDKLPHLQHVFVVGGEAESGTNIINYDEAAKQESTRLDIEWMDKKDGFLLHYTSGSTGTPKGVL
HVHEAMIQQYQTGKWVLDLKEEDIYWCTADPGWVTGTVYGIFAPWLNGATNVIVGGRFSPESWYGTIEQLGVNVW
YSAPTAFRMLMGAGDEMAAKYDLTSLRHVLSVGEPLNPEVIRWGHKVFNKRIHDTWWMTETGSQLICNYPCMDIK
PGSMGKPIPGVEAAIVDNQGNELPPYRMGNLAIKKGWPSMMHTIWNNPEKYESYFMPGGWYVSGDSAYMDEEGYF
WFQGRVDDVIMTSGERVGPFEVESKLVEHPAIAEAGVIGKPDPVRGEIIKAFIALREGFEPSDKLKEEIRLFVKQ
GLAAHAAPREIEFKDKLPKTRSGKIMRRVLKAWELNLPAGDLSTMED:
MEHHKTYHSANIKTATGSLLIEGPVSPEDLAGYEFHKDLTAFRPPREQHEALVDIAGLPEGRIIIARDGRTIVGY
VTYLYPDPLERWSEGNMEDLIELGAIEVAPDYRGCAVGKTLLTVSMMDEQMENYIVMTT**Q**YYWHWDLKGMKKDVW
EYRKIMEKMMNAGGLVWFATDEPEISSHPANCLMARIGKNVSQESIEQFDRLRFYHRYMY

Complex: AcsA•AcuA H139A *B. subtilis* UniProt: P39062, P39065
MNLKALPAIEGDHNLKNYEETYRHFDWAEAEKHFSWHETGKLNAAYEAIDRHAESFRKNKVALYYKDAKRDEKYT
FKEMKEESNRAGNVLRRYGNVEKGDRVFIFMPRSPELYFIMLGAIKIGAIAGPLFEAFMEGAVKDRLENSEAKVV
VTTPELLERIPVDKLPHLQHVFVVGGEAESGTNIINYDEAAKQESTRLDIEWMDKKDGFLLHYTSGSTGTPKGVL
HVHEAMIQQYQTGKWVLDLKEEDIYWCTADPGWVTGTVYGIFAPWLNGATNVIVGGRFSPESWYGTIEQLGVNVW
YSAPTAFRMLMGAGDEMAAKYDLTSLRHVLSVGEPLNPEVIRWGHKVFNKRIHDTWWMTETGSQLICNYPCMDIK
PGSMGKPIPGVEAAIVDNQGNELPPYRMGNLAIKKGWPSMMHTIWNNPEKYESYFMPGGWYVSGDSAYMDEEGYF
WFQGRVDDVIMTSGERVGPFEVESKLVEHPAIAEAGVIGKPDPVRGEIIKAFIALREGFEPSDKLKEEIRLFVKQ
GLAAHAAPREIEFKDKLPKTRSGKIMRRVLKAWELNLPAGDLSTMED:
MEHHKTYHSANIKTATGSLLIEGPVSPEDLAGYEFHKDLTAFRPPREQHEALVDIAGLPEGRIIIARDGRTIVGY
VTYLYPDPLERWSEGNMEDLIELGAIEVAPDYRGCAVGKTLLTVSMMDEQMENYIVMTTQYYW**A**WDLKGMKKDVW
EYRKIMEKMMNAGGLVWFATDEPEISSHPANCLMARIGKNVSQESIEQFDRLRFYHRYMY

Complex: AcsA•AcuA W140A *B. subtilis* UniProt: P39062, P39065
MNLKALPAIEGDHNLKNYEETYRHFDWAEAEKHFSWHETGKLNAAYEAIDRHAESFRKNKVALYYKDAKRDEKYT
FKEMKEESNRAGNVLRRYGNVEKGDRVFIFMPRSPELYFIMLGAIKIGAIAGPLFEAFMEGAVKDRLENSEAKVV
VTTPELLERIPVDKLPHLQHVFVVGGEAESGTNIINYDEAAKQESTRLDIEWMDKKDGFLLHYTSGSTGTPKGVL
HVHEAMIQQYQTGKWVLDLKEEDIYWCTADPGWVTGTVYGIFAPWLNGATNVIVGGRFSPESWYGTIEQLGVNVW

YSAPTAFRMLMGAGDEMAAKYDLTSLRHVLSVGEPLNPEVIRWGHKVFNKRIHDTWWMTETGSQLICNYPCMDIK
PGSMGKPIPGVEAAIVDNQGNELPPYRMGNLAIKKGWPSMMHTIWNNPEKYESYFMPGGWYVSGDSAYMDEEGYF
WFQGRVDDVIMTSGERVGPFEVESKLVEHPAIAEAGVIGKPDPVRGEIIKAFIALREGFEPSDKLKEEIRLFVKQ
GLAAHAAPREIEFKDKLPKTRSGKIMRRVLKAWELNLPAGDLSTMED:
MEHHKTYHSANIKTATGSLLIEGPVSPEDLAGYEFHKDLTAFRPPREQHEALVDIAGLPEGRIIIARDGRTIVGY
VTYLYPDPLERWSEGNMEDLIELGAIEVAPDYRGCAVGKTLLTVSMMDEQMENYIVMTTQYYWH**A**DLKGMKKDVW
EYRKIMEKMMNAGGLVWFATDEPEISSHPANCLMARIGKNVSQESIEQFDRLRFYHRYMY

Complex: AcsA•AcuA W140F *B. subtilis* UniProt: P39062, P39065
MNLKALPAIEGDHNLKNYEETYRHFDWAEAEKHFSWHETGKLNAAYEAIDRHAESFRKNKVALYYKDAKRDEKYT
FKEMKEESNRAGNVLRRYGNVEKGDRVFIFMPRSPELYFIMLGAIKIGAIAGPLFEAFMEGAVKDRLENSEAKVV
VTTPELLERIPVDKLPHLQHVFVVGGEAESGTNIINYDEAAKQESTRLDIEWMDKKDGFLLHYTSGSTGTPKGVL
HVHEAMIQQYQTGKWVLDLKEEDIYWCTADPGWVTGTVYGIFAPWLNGATNVIVGGRFSPESWYGTIEQLGVNVW
YSAPTAFRMLMGAGDEMAAKYDLTSLRHVLSVGEPLNPEVIRWGHKVFNKRIHDTWWMTETGSQLICNYPCMDIK
PGSMGKPIPGVEAAIVDNQGNELPPYRMGNLAIKKGWPSMMHTIWNNPEKYESYFMPGGWYVSGDSAYMDEEGYF
WFQGRVDDVIMTSGERVGPFEVESKLVEHPAIAEAGVIGKPDPVRGEIIKAFIALREGFEPSDKLKEEIRLFVKQ
GLAAHAAPREIEFKDKLPKTRSGKIMRRVLKAWELNLPAGDLSTMED:
MEHHKTYHSANIKTATGSLLIEGPVSPEDLAGYEFHKDLTAFRPPREQHEALVDIAGLPEGRIIIARDGRTIVGY
VTYLYPDPLERWSEGNMEDLIELGAIEVAPDYRGCAVGKTLLTVSMMDEQMENYIVMTTQYYWH**F**DLKGMKKDVW
EYRKIMEKMMNAGGLVWFATDEPEISSHPANCLMARIGKNVSQESIEQFDRLRFYHRYMY

Complex: AcsA•AcuA E85Q E97Q *B. subtilis* UniProt: P39062, P39065
MNLKALPAIEGDHNLKNYEETYRHFDWAEAEKHFSWHETGKLNAAYEAIDRHAESFRKNKVALYYKDAKRDEKYT
FKEMKEESNRAGNVLRRYGNVEKGDRVFIFMPRSPELYFIMLGAIKIGAIAGPLFEAFMEGAVKDRLENSEAKVV
VTTPELLERIPVDKLPHLQHVFVVGGEAESGTNIINYDEAAKQESTRLDIEWMDKKDGFLLHYTSGSTGTPKGVL
HVHEAMIQQYQTGKWVLDLKEEDIYWCTADPGWVTGTVYGIFAPWLNGATNVIVGGRFSPESWYGTIEQLGVNVW
YSAPTAFRMLMGAGDEMAAKYDLTSLRHVLSVGEPLNPEVIRWGHKVFNKRIHDTWWMTETGSQLICNYPCMDIK
PGSMGKPIPGVEAAIVDNQGNELPPYRMGNLAIKKGWPSMMHTIWNNPEKYESYFMPGGWYVSGDSAYMDEEGYF
WFQGRVDDVIMTSGERVGPFEVESKLVEHPAIAEAGVIGKPDPVRGEIIKAFIALREGFEPSDKLKEEIRLFVKQ
GLAAHAAPREIEFKDKLPKTRSGKIMRRVLKAWELNLPAGDLSTMED:
MEHHKTYHSANIKTATGSLLIEGPVSPEDLAGYEFHKDLTAFRPPREQHEALVDIAGLPEGRIIIARDGRTIVGY
VTYLYPDPL**Q**RWSEGNMEDLI**Q**LGAIEVAPDYRGCAVGKTLLTVSMMDEQMENYIVMTTEYYWHWDLKGMKKDVW
EYRKIMEKMMNAGGLVWFATDEPEISSHPANCLMARIGKNVSQESIEQFDRLRFYHRYMY

Complex: AcsA•AcuA E97Q E135Q *B. subtilis* UniProt: P39062, P39065
MNLKALPAIEGDHNLKNYEETYRHFDWAEAEKHFSWHETGKLNAAYEAIDRHAESFRKNKVALYYKDAKRDEKYT
FKEMKEESNRAGNVLRRYGNVEKGDRVFIFMPRSPELYFIMLGAIKIGAIAGPLFEAFMEGAVKDRLENSEAKVV
VTTPELLERIPVDKLPHLQHVFVVGGEAESGTNIINYDEAAKQESTRLDIEWMDKKDGFLLHYTSGSTGTPKGVL
HVHEAMIQQYQTGKWVLDLKEEDIYWCTADPGWVTGTVYGIFAPWLNGATNVIVGGRFSPESWYGTIEQLGVNVW
YSAPTAFRMLMGAGDEMAAKYDLTSLRHVLSVGEPLNPEVIRWGHKVFNKRIHDTWWMTETGSQLICNYPCMDIK
PGSMGKPIPGVEAAIVDNQGNELPPYRMGNLAIKKGWPSMMHTIWNNPEKYESYFMPGGWYVSGDSAYMDEEGYF
WFQGRVDDVIMTSGERVGPFEVESKLVEHPAIAEAGVIGKPDPVRGEIIKAFIALREGFEPSDKLKEEIRLFVKQ
GLAAHAAPREIEFKDKLPKTRSGKIMRRVLKAWELNLPAGDLSTMED:
MEHHKTYHSANIKTATGSLLIEGPVSPEDLAGYEFHKDLTAFRPPREQHEALVDIAGLPEGRIIIARDGRTIVGY
VTYLYPDPLERWSEGNMEDLI**Q**LGAIEVAPDYRGCAVGKTLLTVSMMDEQMENYIVMTT**Q**YYWHWDLKGMKKDVW
EYRKIMEKMMNAGGLVWFATDEPEISSHPANCLMARIGKNVSQESIEQFDRLRFYHRYMY

Complex: AcsA K549Q•AcuA *B. subtilis* UniProt: P39062, P39065
MNLKALPAIEGDHNLKNYEETYRHFDWAEAEKHFSWHETGKLNAAYEAIDRHAESFRKNKVALYYKDAKRDEKYT
FKEMKEESNRAGNVLRRYGNVEKGDRVFIFMPRSPELYFIMLGAIKIGAIAGPLFEAFMEGAVKDRLENSEAKVV
VTTPELLERIPVDKLPHLQHVFVVGGEAESGTNIINYDEAAKQESTRLDIEWMDKKDGFLLHYTSGSTGTPKGVL
HVHEAMIQQYQTGKWVLDLKEEDIYWCTADPGWVTGTVYGIFAPWLNGATNVIVGGRFSPESWYGTIEQLGVNVW
YSAPTAFRMLMGAGDEMAAKYDLTSLRHVLSVGEPLNPEVIRWGHKVFNKRIHDTWWMTETGSQLICNYPCMDIK
PGSMGKPIPGVEAAIVDNQGNELPPYRMGNLAIKKGWPSMMHTIWNNPEKYESYFMPGGWYVSGDSAYMDEEGYF
WFQGRVDDVIMTSGERVGPFEVESKLVEHPAIAEAGVIGKPDPVRGEIIKAFIALREGFEPSDKLKEEIRLFVKQ
GLAAHAAPREIEFKDKLPKTRSG**Q**IMRRVLKAWELNLPAGDLSTMED:
MEHHKTYHSANIKTATGSLLIEGPVSPEDLAGYEFHKDLTAFRPPREQHEALVDIAGLPEGRIIIARDGRTIVGY
VTYLYPDPLERWSEGNMEDLIELGAIEVAPDYRGCAVGKTLLTVSMMDEQMENYIVMTTEYYWHWDLKGMKKDVW
EYRKIMEKMMNAGGLVWFATDEPEISSHPANCLMARIGKNVSQESIEQFDRLRFYHRYMY

Complex: AcsA K549R•AcuA *B. subtilis* UniProt: P39062, P39065
MNLKALPAIEGDHNLKNYEETYRHFDWAEAEKHFSWHETGKLNAAYEAIDRHAESFRKNKVALYYKDAKRDEKYT
FKEMKEESNRAGNVLRRYGNVEKGDRVFIFMPRSPELYFIMLGAIKIGAIAGPLFEAFMEGAVKDRLENSEAKVV

VTTPELLERIPVDKLPHLQHVFVVGGEAESGTNIINYDEAAKQESTRLDIEWMDKKDGFLLHYTSGSTGTPKGVL
HVHEAMIQQYQTGKWVLDLKEEDIYWCTADPGWVTGTVYGIFAPWLNGATNVIVGGRFSPESWYGTIEQLGVNVW
YSAPTAFRMLMGAGDEMAAKYDLTSLRHVLSVGEPLNPEVIRWGHKVFNKRIHDTWWMTETGSQLICNYPCMDIK
PGSMGKPIPGVEAAIVDNQGNELPPYRMGNLAIKKGWPSMMHTIWNNPEKYESYFMPGGWYVSGDSAYMDEEGYF
WFQGRVDDVIMTSGERVGPFEVESKLVEHPAIAEAGVIGKPDPVRGEIIKAFIALREGFEPSDKLKEEIRLFVKQ
GLAAHAAPREIEFKDKLPKTRSG**R**IMRRVLKAWELNLPAGDLSTMED:
MEHHKTYHSANIKTATGSLLIEGPVSPEDLAGYEFHKDLTAFRPPREQHEALVDIAGLPEGRIIIARDGRTIVGY
VTYLYPDPLERWSEGNMEDLIELGAIEVAPDYRGCAVGKTLLTVSMMDEQMENYIVMTTEYYWHWDLKGMKKDVW
EYRKIMEKMMNAGGLVWFATDEPEISSHPANCLMARIGKNVSQESIEQFDRLRFYHRYMY

Complex: AcsA E473A•AcuA *B. subtilis* UniProt: P39062, P39065
MNLKALPAIEGDHNLKNYEETYRHFDWAEAEKHFSWHETGKLNAAYEAIDRHAESFRKNKVALYYKDAKRDEKYT
FKEMKEESNRAGNVLRRYGNVEKGDRVFIFMPRSPELYFIMLGAIKIGAIAGPLFEAFMEGAVKDRLENSEAKVV
VTTPELLERIPVDKLPHLQHVFVVGGEAESGTNIINYDEAAKQESTRLDIEWMDKKDGFLLHYTSGSTGTPKGVL
HVHEAMIQQYQTGKWVLDLKEEDIYWCTADPGWVTGTVYGIFAPWLNGATNVIVGGRFSPESWYGTIEQLGVNVW
YSAPTAFRMLMGAGDEMAAKYDLTSLRHVLSVGEPLNPEVIRWGHKVFNKRIHDTWWMTETGSQLICNYPCMDIK
PGSMGKPIPGVEAAIVDNQGNELPPYRMGNLAIKKGWPSMMHTIWNNPEKYESYFMPGGWYVSGDSAYMDEEGYF
WFQGRVDDVIMTSGERVGPFEV**A**SKLVEHPAIAEAGVIGKPDPVRGEIIKAFIALREGFEPSDKLKEEIRLFVKQ
GLAAHAAPREIEFKDKLPKTRSGKIMRRVLKAWELNLPAGDLSTMED:
MEHHKTYHSANIKTATGSLLIEGPVSPEDLAGYEFHKDLTAFRPPREQHEALVDIAGLPEGRIIIARDGRTIVGY
VTYLYPDPLERWSEGNMEDLIELGAIEVAPDYRGCAVGKTLLTVSMMDEQMENYIVMTTEYYWHWDLKGMKKDVW
EYRKIMEKMMNAGGLVWFATDEPEISSHPANCLMARIGKNVSQESIEQFDRLRFYHRYMY

Complex: AcsA V477A•AcuA *B. subtilis* UniProt: P39062, P39065
MNLKALPAIEGDHNLKNYEETYRHFDWAEAEKHFSWHETGKLNAAYEAIDRHAESFRKNKVALYYKDAKRDEKYT
FKEMKEESNRAGNVLRRYGNVEKGDRVFIFMPRSPELYFIMLGAIKIGAIAGPLFEAFMEGAVKDRLENSEAKVV
VTTPELLERIPVDKLPHLQHVFVVGGEAESGTNIINYDEAAKQESTRLDIEWMDKKDGFLLHYTSGSTGTPKGVL
HVHEAMIQQYQTGKWVLDLKEEDIYWCTADPGWVTGTVYGIFAPWLNGATNVIVGGRFSPESWYGTIEQLGVNVW
YSAPTAFRMLMGAGDEMAAKYDLTSLRHVLSVGEPLNPEVIRWGHKVFNKRIHDTWWMTETGSQLICNYPCMDIK
PGSMGKPIPGVEAAIVDNQGNELPPYRMGNLAIKKGWPSMMHTIWNNPEKYESYFMPGGWYVSGDSAYMDEEGYF
WFQGRVDDVIMTSGERVGPFEVESKL**A**EHPAIAEAGVIGKPDPVRGEIIKAFIALREGFEPSDKLKEEIRLFVKQ
GLAAHAAPREIEFKDKLPKTRSGKIMRRVLKAWELNLPAGDLSTMED:
MEHHKTYHSANIKTATGSLLIEGPVSPEDLAGYEFHKDLTAFRPPREQHEALVDIAGLPEGRIIIARDGRTIVGY
VTYLYPDPLERWSEGNMEDLIELGAIEVAPDYRGCAVGKTLLTVSMMDEQMENYIVMTTEYYWHWDLKGMKKDVW
EYRKIMEKMMNAGGLVWFATDEPEISSHPANCLMARIGKNVSQESIEQFDRLRFYHRYMY

Complex: AcsA E473A•AcuA *B. subtilis* UniProt: P39062, P39065
MNLKALPAIEGDHNLKNYEETYRHFDWAEAEKHFSWHETGKLNAAYEAIDRHAESFRKNKVALYYKDAKRDEKYT
FKEMKEESNRAGNVLRRYGNVEKGDRVFIFMPRSPELYFIMLGAIKIGAIAGPLFEAFMEGAVKDRLENSEAKVV
VTTPELLERIPVDKLPHLQHVFVVGGEAESGTNIINYDEAAKQESTRLDIEWMDKKDGFLLHYTSGSTGTPKGVL
HVHEAMIQQYQTGKWVLDLKEEDIYWCTADPGWVTGTVYGIFAPWLNGATNVIVGGRFSPESWYGTIEQLGVNVW
YSAPTAFRMLMGAGDEMAAKYDLTSLRHVLSVGEPLNPEVIRWGHKVFNKRIHDTWWMTETGSQLICNYPCMDIK
PGSMGKPIPGVEAAIVDNQGNELPPYRMGNLAIKKGWPSMMHTIWNNPEKYESYFMPGGWYVSGDSAYMDEEGYF
WFQGRVDDVIMTSGERVGPFEV**A**SKLVEHPAIAEAGVIGKPDPVRGEIIKAFIALREGFEPSDKLKEEIRLFVKQ
GLAAHAAPREIEFKDKLPKTRSGKIMRRVLKAWELNLPAGDLSTMED:
MEHHKTYHSANIKTATGSLLIEGPVSPEDLAGYEFHKDLTAFRPPREQHEALVDIAGLPEGRIIIARDGRTIVGY
VTYLYPDPLERWSEGNMEDLIELGAIEVAPDYRGCAVGKTLLTVSMMDEQMENYIVMTTEYYWHWDLKGMKKDVW
EYRKIMEKMMNAGGLVWFATDEPEISSHPANCLMARIGKNVSQESIEQFDRLRFYHRYMY

Supplementary Figure 3: Sequences of *B. subtilis* AcsA (UniProt: P39062) and *B. subtilis* AcuA (UniProt: P39065) used for AlphaFold2 structure predictions. AlphaFold2 version 2.2.4 was used to perform structure predictions of the AcsA monomer, the AcsA dimer, and of the complex of AcsA•AcuA. For the predicted structures of the complexes of mutated AcsA or AcuA, the sequences were used carrying the alterations from the wildtype sequences highlighted in red. For the complexes, the sequences preceding the colon correspond to AcsA, the sequence following the colon correspond to AcuA or the indicated single or double mutants thereof.

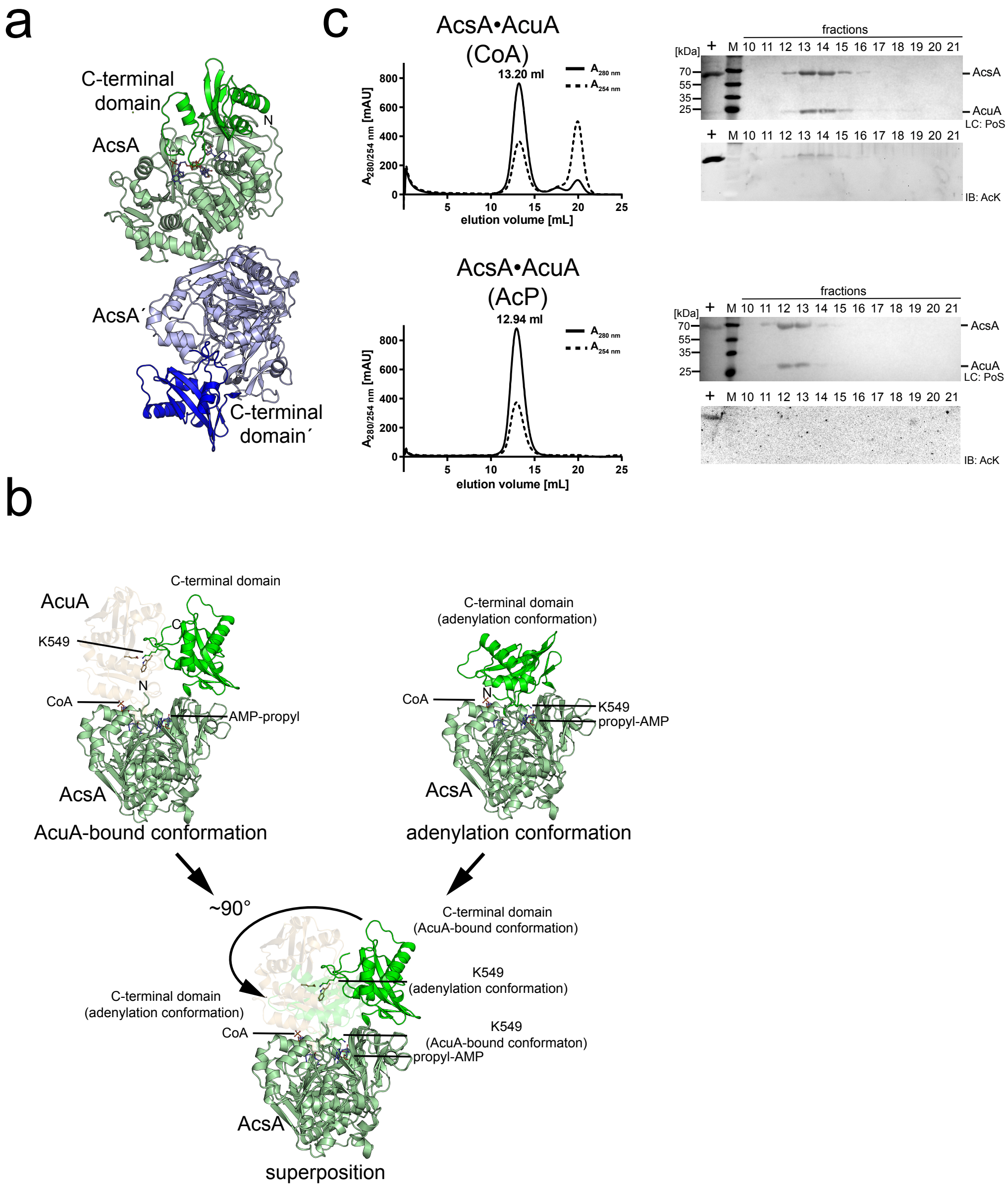

Supplementary Figure 4: AlphaFold2 structure prediction of AcsA and AcuA suggest formation of a AcsA•AcsA homodimer.

- **a** AlphaFold2 was used to predict the structure of a $AcsA_2$ homodimer. The two AcsA monomers interact in a tail-to-tail fashion creating an apparent twofold symmetry. Both AcsA molecules are in the conformation for the adenylation reaction, i.e. formation of acetyl-AMP and PP_i from acetate and ATP.
- **b** AcuA binding to AcsA and position of the AcsA C-terminal domain in the adenylation conformation are mutually exclusive. Binding of AcuA to AcsA results in a huge, app. 90° conformational change of the AcsA C-terminal domain, stabilizing AcsA in conformation not observed before. Acetylation of K549 in AcsA by AcuA results in weakening of the AcsA•AcuA complex. The C-terminal domain of AcsA is capable to displace AcuA from AcsA upon adapting the adenylation conformation.
- **c** Analytical-size exclusion chromatography (SEC) experiments show that AcsA and AcuA form a stable complex in presence of coenzyme A (CoA) (upper panel) or acetyl-phosphate (AcP) (lower panel). As described for AcsA•AcuA, the elution volume suggests that AcsA and AcuA form an apparent heterotetramer composed of two AcsA•AcuA heterodimers. PoS: Ponceaut S-red staining was done as loading control. Immunoblotting with anti-acetyl-lysine antibody (anti-AcK AB) was done to stain for acetylated AcsA (IB: AcK). The lane labelled with + shows the acetylated *B. subtilis* AcsA loaded as technical control for the immunoblot, the lane labelled with M represents the protein molecular weight marker. Source data are provided as Source Data file and in Supplementary Fig. 16.

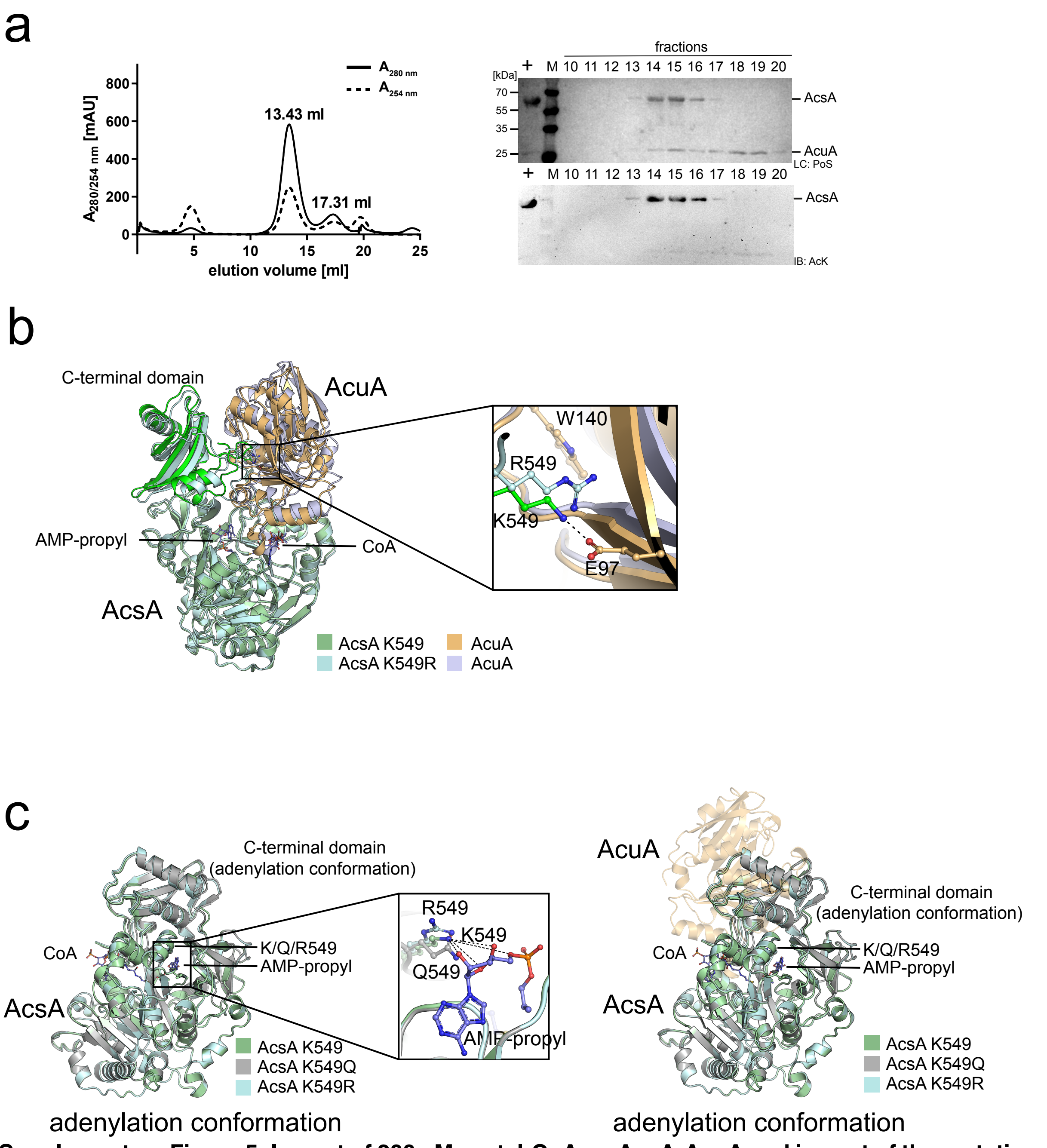

Supplementary Figure 5: Impact of 200 μM acetyl-CoA on AcsA•AcuA and impact of the mutation K549R in AcsA on the structure of AcsA and AcsA•AcuA by AlphaFold2.

a Treatment of AcsA•AcuA with 200 μM acetyl-CoA results in acetylation of AcsA and dissociation of the complex. PoS: Ponceau S-red staining was done as loading control. Immunoblotting with anti-acetyl-lysine antibody (anti-AcK AB) was done to stain for acetylated AcsA (IB: AcK). The lane labelled with + shows the acetylated *B. subtilis* AcsA loaded as technical control for the immunoblot, the lane labelled with M represents the protein molecular weight marker. Source data are provided as Source Data file and in Supplementary Fig. 17.

b AlphaFold2 structure predictions of the complex of AcsA K549R•AcuA suggest a conformation similar to wildtype AcsA•AcuA incompatible to catalyse the first or second half-reaction. The superposition was done by PyMOL[1].

c AcsA K549Q, AcsA K549R and wildtype AcsA are in the adenylation conformation as shown by AlphaFold2 structure prediction. Superposition of AcsA K549Q, AcsA K549R to AcsA•AcuA indicates the adenylation conformation being incompatible with AcuA binding. The figure and superposition were done using PyMOL[1].

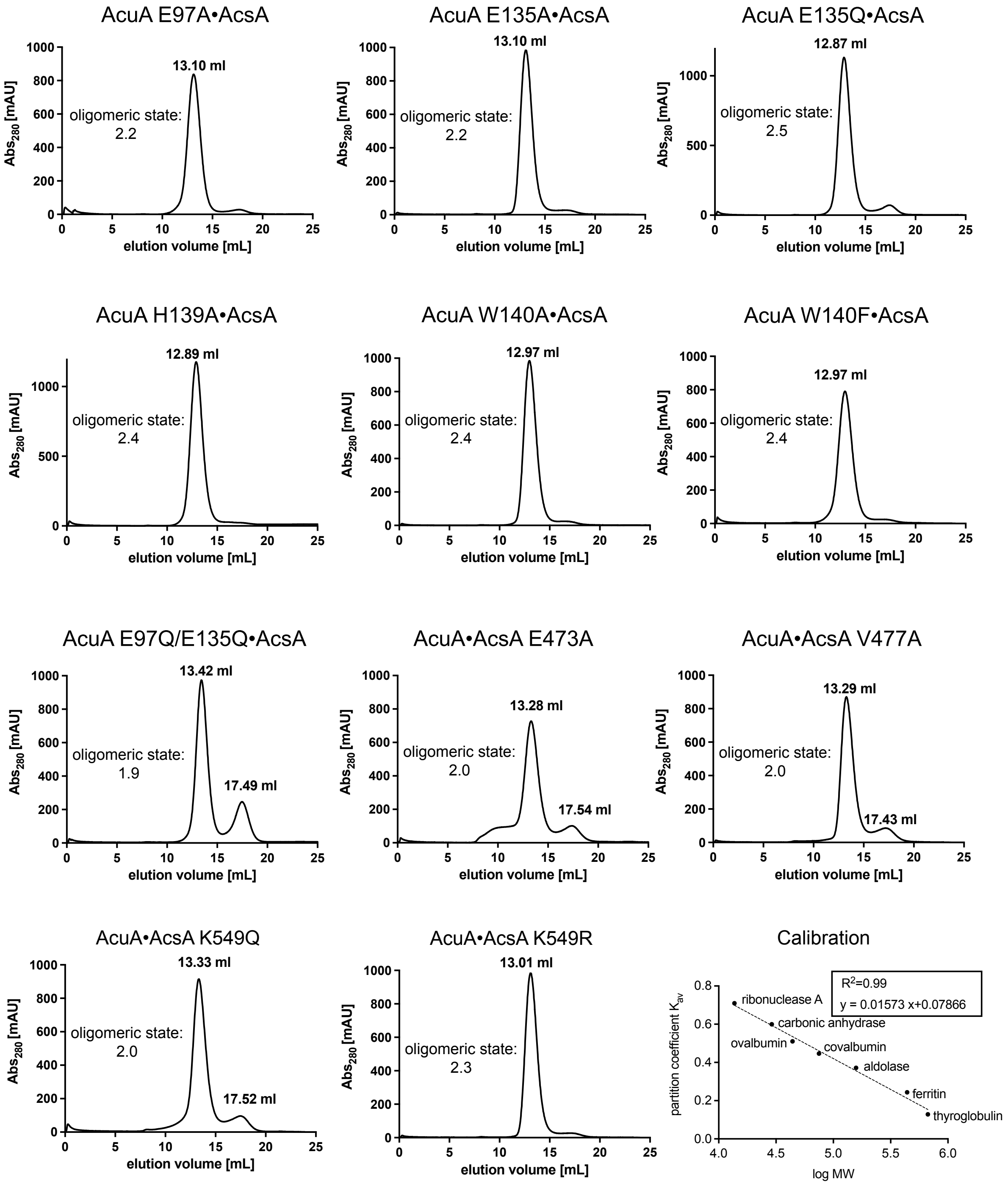

Supplementary Figure 6: Analytical size exclusion chromatography (SEC) experiments of complexes between mutants of AcuA or mutants of AcsA with their wildtype counterparts. All experiments were conducted on a calibrated Superdex 200 10/300 GL column with equimolar concentrations of AcuA and AcsA. The calculated oligomeric states are indicated. The complex of the double mutant AcuA E97Q/E135Q with wildtype AcsA and of wildtype AcuA with AcsA E473A, AcsA V477A or AcsA K549Q show free AcuA eluting from the column indicating the mutation impairing affinity of AcsA to AcuA. Source data are provided as Source Data file.

```
                                                 1        10
B. subtilis                ...............................MNLKALPAIEGD....HNLKN........
Chloroflexota bacterium    ...............MSSNLSFEHTDNILIEERLFPPPEDIVK....NANITAYMKSKGF
S. typhimurium             ............................MSQTHKHAIPANIAD....RCLINPEQ.....
E. coli                    ............................MSQIHKHTIPANIAD....RCLINPQQ.....
M. tuberculosis            .......................MSESTPEVSSSYPPPAHFAE....HANA.........
H. sapiens                 ......MGLPEERVRSGSGSRGQEEAGAGGRARSWSPPPEVSR....SAHVPSLQR....
X. oryzae                  ..............................MADVYPVDPAFAA....DARVTREQ.....
B. migulanus               MLNSSKSILIHAQNKNGTHEEEQYLFAVNNTKAEYPRDKTIHQLFEEQVSKRPNNVAIVC
P. pyralis                 ..................................MEDAKNIKKGPAP.............

                              α1            α2                                α3
                              20            30                           40
B. subtilis                ..YEETYRHFD......WAEAEK........................HFSWHETGKLNAA
Chloroflexota bacterium    DDYEAFYRWSLANRFEFWNDMAKELHWFEPWK...STFEWTDKP...FFKWFTDGKFNIA
S. typhimurium             ..YETKYKQSINDPDTFWGEQGKILDWITPYQ...KVKNTSFAPGNVSIKWYEDGTLNLA
E. coli                    ..YEAMYQQSINVPDTFWGEQGKILDWIKPYQ...KVKNTSFAPGNVSIKWYEDGTLNLA
M. tuberculosis            ..RAELYREAEEDRLAFWAKQANRLSWTTPFT...EVLDWSGAP...FAKWFVGGELNVA
H. sapiens                 ..YRELHRRSVEEPREFWGDIAKEFYWKTPCPGPFLRYNFDVTKGKIFIEWMKGATTNIC
X. oryzae                  ..YAALYRESIEHPEQFWGKAAQRLEWFKQPT...QVKDVSYALDDFHIRWFGDGELNAS
B. migulanus               ENEQLTYHELNVKANQLARIFIEKGIGKDTLVGIMMEKSIDLFIGILAVLKAGGAYVPID
P. pyralis                 ...............................................FYPLEDGTAGEQL

                           α4   α5          β1           β2                    α6
                              50          60          70                          80
B. subtilis                YEAIDRHAESFR.KNKVALYYKDAK...RDEKYTFK...................EMKEE
Chloroflexota bacterium    YNCLDRYMGTP.IEDKVAFYWEGDD..GSSRAYTYK...................EMYVL
S. typhimurium             ANCLDRHLQEN..GDRTAIIWEGDD.TSQSKHISYR...................ELHRD
E. coli                    ANCLDRHLQEN..GDRTAIIWEGDD.ASQSKHISYK...................ELHRD
M. tuberculosis            YNCVDRHVEAG.HGDRVAIHWEGEP.VGDRRTLTYS...................DLLAE
H. sapiens                 YNVLDRNVHEKKLGDKVAFYWEGNE.PGETTQITYH...................QLLVQ
X. oryzae                  VNCLDRQLATR..GDKTALLFEPDSPDAASYPVTYR...................QLYER
B. migulanus               IEYPKERIQYILDDSQARMLLTQKHLVHLIHNIQFNGQVEIFEEDTIKIREGTNLHVPSK
P. pyralis                 HKAMKRYALVP.....GTIAFTDAH...IEVNITYA...................EYFEM

                                             TT     β3               α7               β4     TT
                                   90        100        110        120        130
B. subtilis                SNRAGNVLRRYGNVEKGDRVFIFMPRSP......ELYFIMLGAIKIGAIAGPLFEAFMEG
Chloroflexota bacterium    TNRVAKVLQNQG.VKKGDRVAIYMPMIP......EMAASVLACARLGAPHMVVFGGFAAS
S. typhimurium             VCRFANTLLDLG.IKKGDVVAIYMPMVP......EAAVAMLACARIGAVHSVIFGGFSPE
E. coli                    VCRFANTLLELG.IKKGDVVAIYMPMVP......EAAVAMLACARIGAVHSVIFGGFSPE
M. tuberculosis            VSKAANALTDLG.LVAGDRVAIYLPLIP......EAVIAMLACARLGIMHSVVFGGFTAA
H. sapiens                 VCQFSNVLRKQG.IQKGDRVAIYMPMIP......ELVVAMLACARIGALHSIVFAGFSSE
X. oryzae                  VCKLGNALRNLG.VKKGDRVTIYLPMIV......DAAVAMLACARIGAVHSVVFGGFAAN
B. migulanus               STDLAYVIYTSGTTGNPKGTMLEHKGISNLKVFFENSLNVTEKDRIGQFASISFDASVWE
P. pyralis                 SVRLAEAMKRYG.LNTNHRIVVCSENSL......QFFMPVLGALFIGVAVAPANDIYNER

                              α8           β5       α9               η1   T            T    β6
                             140         150                       160                 170
B. subtilis                AVKDRLENSEAKVVVTTPE.......LLER..IPVDKLP.............HLQHVFVV
Chloroflexota bacterium    SLRDRMNDCDAKVLITADG.......GYRGGKVIELKKIADEAVAETPTI....EKVFVQ
S. typhimurium             AVAGRIIDSSSRLVITADE.......GVRAGRSIPLKKNVDDALKNPNVT..SVEHVIVL
E. coli                    AVAGRIIDSNSRLVITSDE.......GVRAGRSIPLKKNVDDALKNPNVT..SVEHVVVL
M. tuberculosis            ALQARIVDAQAKLLITADG.......QFRRGKPSPLKAAADEALAAIPDC..SVEHVLVV
H. sapiens                 SLCERILDSSCSLLITTDA.......FYRGEKLVNLKELADEALQKCQEKGFPVRCCIVV
X. oryzae                  SIADRVIDCQSKLIITADE.......GLRGGKKIPLKANVDAALKIPGTN..TIETVLVV
B. migulanus               MFMALLTGASLYIILKDTINDFVKFEQYINQKEITVITLPPTYVVHLDPERILSIQTLIT
P. pyralis                 ELLNSMNISQPTVVFVSKK.......GLQKILNVQKKLPIIQKIIIMDSK.........T

                                                         TT β7  α10                TT
                                                         180       190           200
B. subtilis                GGEA...........................ESGTNIINYDEAAKQESTRLD.IEWMDKK
Chloroflexota bacterium    RHTG......................FEVPMAEGRDVYLDVLLNDIPEDTVVPCEPVDSE
S. typhimurium             KRTG......................SDIDWQEGRDLWWRDLIEKASPEHQP..EAMNAE
E. coli                    KRTG......................GKIDWQEGRDLWWHDLVEQASDQHQA..EEMNAE
M. tuberculosis            RRTG......................IEMAWSEGRDLWWHHVVGSASPAHTP..EPFDSE
H. sapiens                 KHLGRAELGMGDSTSQSPPIKRSCPDVQISWNQGIDLWWHELMQEAGDECEP..EWCDAE
X. oryzae                  RHTG......................GAVEMQAPRDRWFHDVVDGQPAECEP..ERMNAE
B. migulanus               AGSATSPSLVNKWKEKVTYINAYGPTETTICATTWVATKETIGHSVPIGAPIQNTQIYIV
P. pyralis                 DYQG..........................FQSMYTFVTSHLPPGFNEYDFVPESFDRDK
```

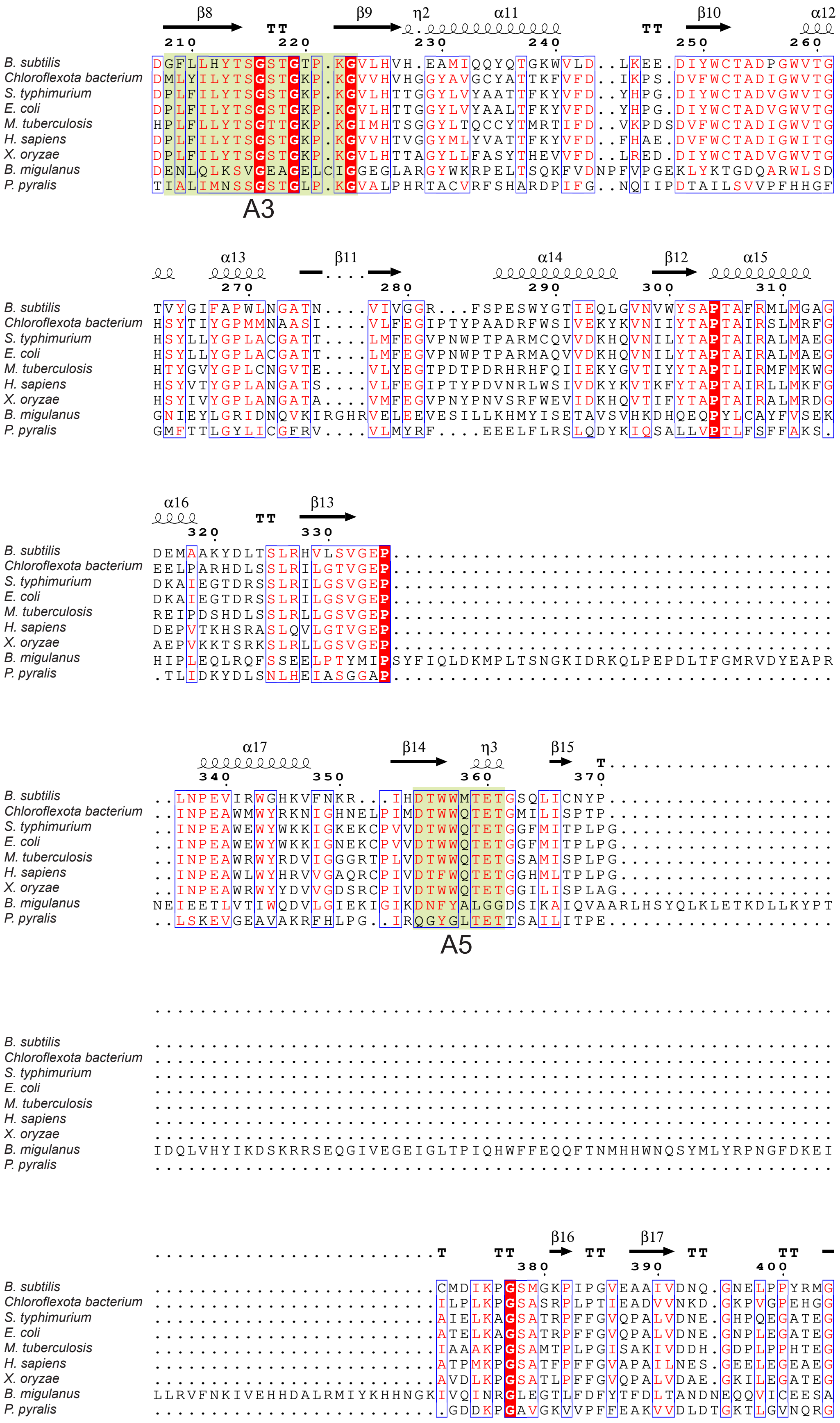
B. subtilis
Chloroflexota bacterium
S. typhimurium
E. coli
M. tuberculosis
H. sapiens
X. oryzae
B. migulanus
P. pyralis
A3
A5

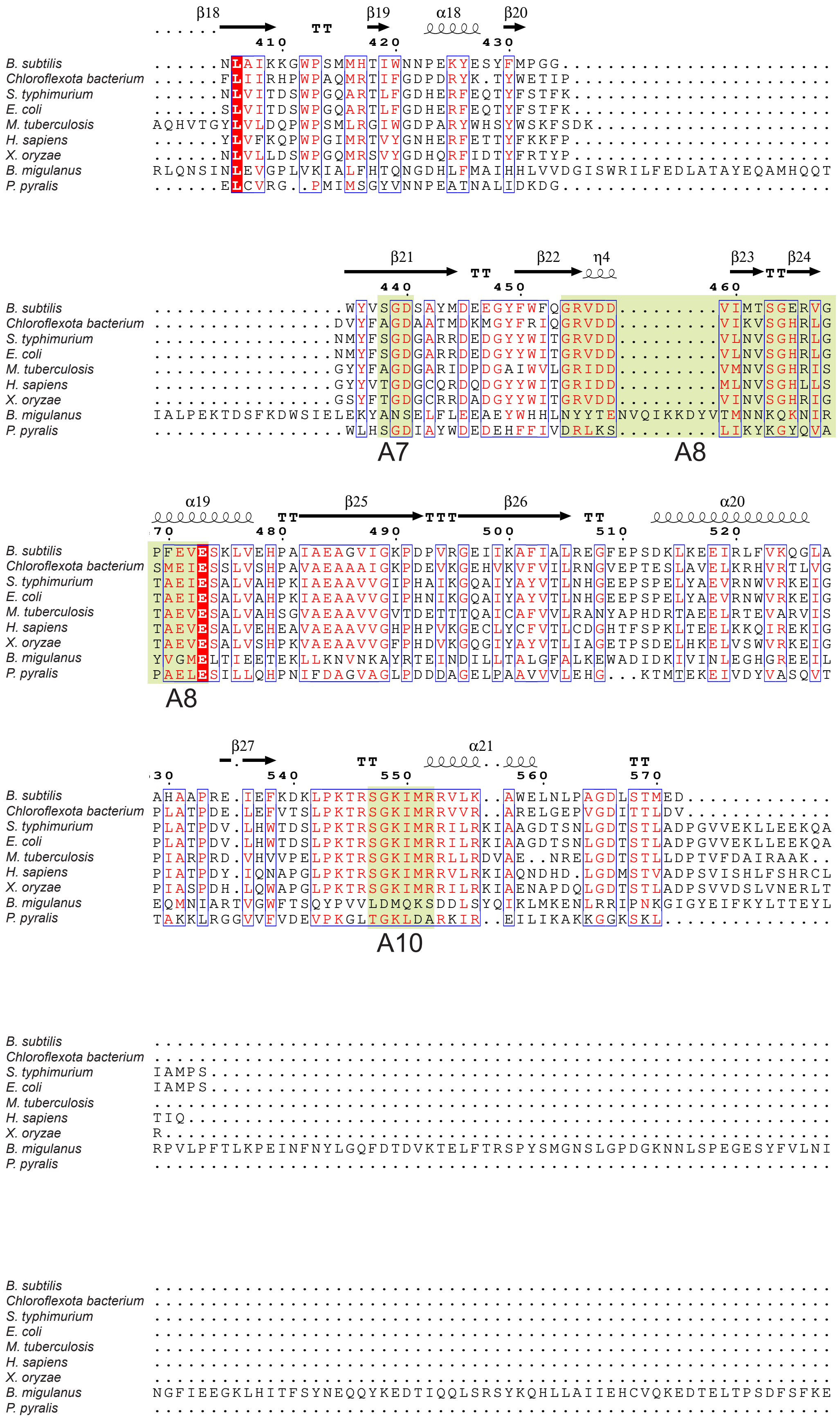

β18 β19 α18 β20
TT
410 420 430
B. subtilis ......NLAIKKGWPSMMHTIWNNPEKYESYFMPGG........................
Chloroflexota bacterium ......FLIIRHPWPAQMRTIFGDPDRYK.TYWETIP.......................
S. typhimurium ......NLVITDSWPGQARTLFGDHERFEQTYFSTFK.......................
E. coli ......SLVITDSWPGQARTLFGDHERFEQTYFSTFK.......................
M. tuberculosis AQHVTGYLVLDQPWPSMLRGIWGDPARYWHSYWSKFSDK.....................
H. sapiens ......YLVFKQPWPGIMRTVYGNHERFETTYFKKFP.......................
X. oryzae ......NLVLLDSWPGQMRSVYGDHQRFIDTYFRTYP.......................
B. migulanus RLQNSINLEVGPLVKIALFHTQNGDHLFMAIHHLVVDGISWRILFEDLATAYEQAMHQQT
P. pyralis ......ELCVRG..PMIMSGYVNNPEATNALIDKDG........................
β21 β22 η4 β23 β24
TT TT
440 450 460
B. subtilis ................WYVSGDSAYMDEEGYFWFQGRVDD.........VIMTSGERVG
Chloroflexota bacterium ................DVYFAGDAATMDKMGYFRIQGRVDD.........VIKVSGHRLG
S. typhimurium ................NMYFSGDGARRDEDGYYWITGRVDD.........VLNVSGHRLG
E. coli ................NMYFSGDGARRDEDGYYWITGRVDD.........VLNVSGHRLG
M. tuberculosis ................GYYFAGDGARIDPDGAIWVLGRIDD.........VMNVSGHRIS
H. sapiens ................GYYVTGDGCQRDQDGYYWITGRIDD.........MLNVSGHLLS
X. oryzae ................GSYFTGDGCRRDADGYYWITGRVDD.........VINVSGHRIG
B. migulanus IALPEKTDSFKDWSIELEKYANSELFLEEAEYWHHLNYYTENVQIKKDYVTMNNKQKNIR
P. pyralis ................WLHSGDIAYWDEDEHFFIVDRLKS.........LIKYKGYQVA
A7 A8
α19 β25 β26 α20
TT TTT TT
470 480 490 500 510 520
B. subtilis PFEVESKLVEHPAIAEAGVIGKPDPVRGEIIKAFIALREGFEPSDKLKEEIRLFVKQGLA
Chloroflexota bacterium SMEIESSLVSHPAVAEAAAIGKPDEVKGEHVKVFVILRNGVEPTESLAVELKRHVRTLVG
S. typhimurium TAEIESALVAHPKIAEAAVVGIPHAIKGQAIYAYVTLNHGEEPSPELYAEVRNWVRKEIG
E. coli TAEIESALVAHPKIAEAAVVGIPHNIKGQAIYAYVTLNHGEEPSPELYAEVRNWVRKEIG
M. tuberculosis TAEVESALVAHSGVAEAAVVGVTDETTTQAICAFVVLRANYAPHDRTAEELRTEVARVIS
H. sapiens TAEVESALVEHEAVAEAAVVGHPHPVKGECLYCFVTLCDGHTFSPKLTEELKKQIREKIG
X. oryzae TAEVESALVSHPKVAEAAVVGFPHDVKGQGIYAYVTLIAGETPSDELHKELVSWVRKEIG
B. migulanus YVGMELTIEETEKLLKNVNKAYRTEINDILLTALGFALKEWADIDKIVINLEGHGREEIL
P. pyralis PAELESILLQHPNIFDAGVAGLPDDDAGELPAAVVVLEHG...KTMTEKEIVDYVASQVT
A8
β27 α21
TT TT
530 540 550 560 570
B. subtilis AHAAPRE.IEFKDKLPKTRSGKIMRRVLK..AWELNLPAGDLSTMED............
Chloroflexota bacterium PLATPDE.LEFVTSLPKTRSGKIMRRVVR..ARELGEPVGDITTLDV............
S. typhimurium PLATPDV.LHWTDSLPKTRSGKIMRRILRKIAAGDTSNLGDTSTLADPGVVEKLLEEKQA
E. coli PLATPDV.LHWTDSLPKTRSGKIMRRILRKIAAGDTSNLGDTSTLADPGVVEKLLEEKQA
M. tuberculosis PIARPRD.VHVVPELPKTRSGKIMRRLLRDVAE..NRELGDTSTLLDPTVFDAIRAAK..
H. sapiens PIATPDY.IQNAPGLPKTRSGKIMRRVLRKIAQNDHD.LGDMSTVADPSVISHLFSHRCL
X. oryzae PIASPDH.LQWAPGLPKTRSGKIMRRILRKIAENAPDQLGDTSTLADPSVVDSLVNERLT
B. migulanus EQMNIARTVGWFTSQYPVVLDMQKSDDLSYQIKLMKENLRRIPNKGIGYEIFKYLTTEYL
P. pyralis TAKKLRGGVVFVDEVPKGLTGKLDARKIR..EILIKAKGGKSKL..............
A10
B. subtilis ....
Chloroflexota bacterium ....
S. typhimurium IAMPS...
E. coli IAMPS...
M. tuberculosis ....
H. sapiens TIQ...
X. oryzae R...
B. migulanus RPVLPFTLKPEINFNYLGQFDTDVKTELFTRSPYSMGNSLGPDGKNNLSPEGESYFVLNI
P. pyralis ....
B. subtilis ....
Chloroflexota bacterium ....
S. typhimurium ....
E. coli ....
M. tuberculosis ....
H. sapiens ....
X. oryzae ....
B. migulanus NGFIEEGKLHITFSYNEQQYKEDTIQQLSRSYKQHLLAIIEHCVQKEDTELTPSDFSFKE
P. pyralis ....

B. subtilis	....
Chloroflexota bacterium	....
S. typhimurium	....
E. coli	....
M. tuberculosis	....
H. sapiens	....
X. oryzae	....
B. migulanus	LELEEMDDIFDLLADSLT
P. pyralis	....

Supplementary Figure 7: Sequence alignment of enzymes of the ANL-superfamily. The alignment was conducted to uncover and highlight conserved regions in *B. subtilis* AcsA (uniport: P39062) and other ANL-enzymes. For AcsA the core sequence motifs A3, A5, A8 and A10 are highlighted. The *B. subtilis* AcsA K549 is highly conserved from mammalian acetyl-CoA synthetases to bacterial enzymes suggesting to play an important functional role (P39062: *B. subtilis* AcsA; A0A535FEC2: acetate-CoA ligase from *Chloroflexota* bacterium; Q8ZKFK6: *Salmonella typhimurium* Acs; P27550: *Escherichia coli* Acs; P9WQD1: *Mycobacterium tuberculosis* AcsA; Q9NR19: *Homo sapiens* AceCS1; Q2NXE2. *Xanthomonas oryzae* Acs; P0C061: *Bacillus migulanus* Gramicidin S synthase I; P08659: *Photinus pyralis* Luciferase). The secondary structure elements are given on the top for *B. subtilis* AcsA. The sequence alignments were conducted with ClustalW and ESPript 3.0 was used to render sequence similarities and information on secondary structure elements derived from the AlphaFold2 structure predictions.

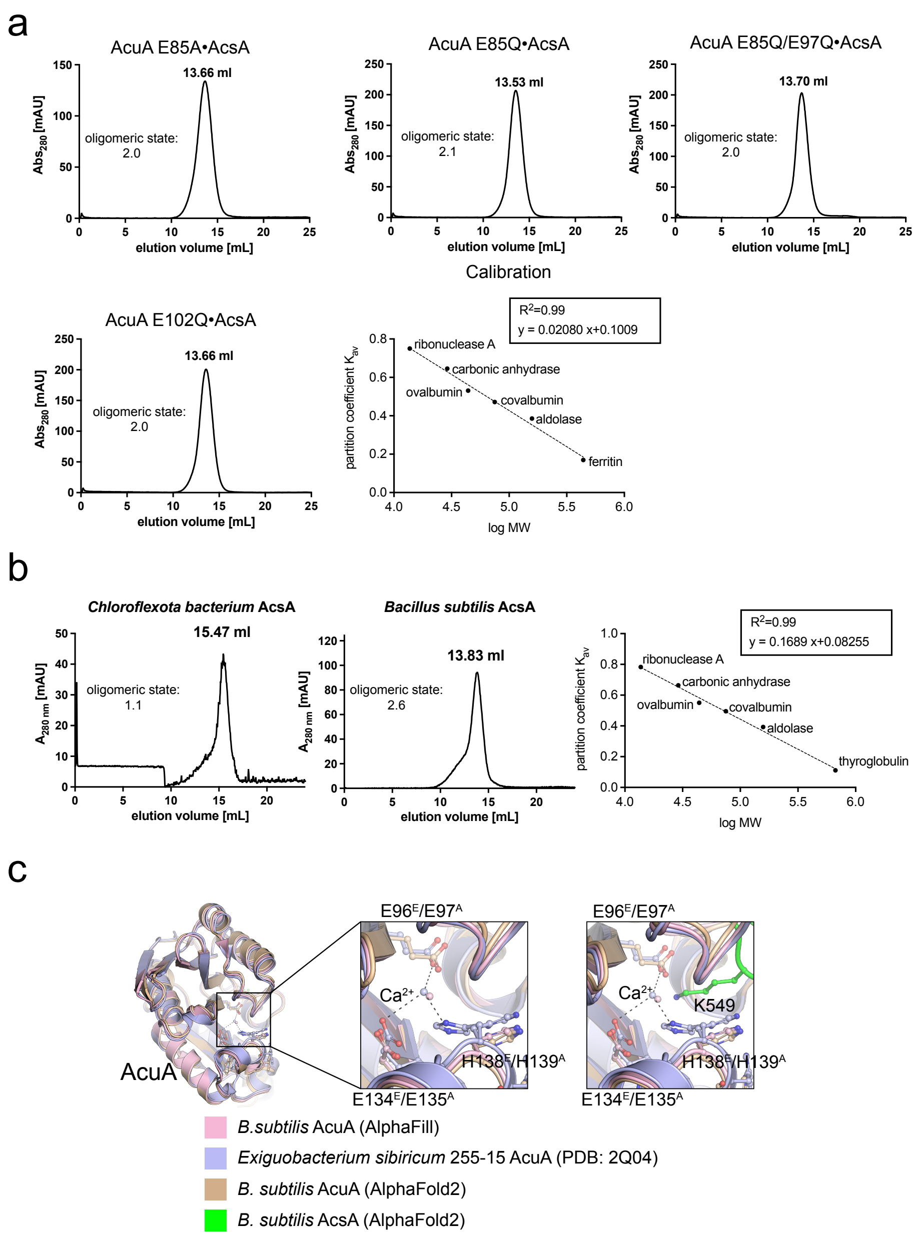

Supplementary Figure 8: The glutamic acids E97, E135 and H139 in *B. subtilis* AcuA could form a binding site for positively-charged ions and the positively-charged amino group of K549 of AcsA.

a Interactions of AcuA mutants with AcsA wildtype analysed by analytical size exclusion chromatography. All experiments were conducted on a calibrated Superdex 200 10/300 GL column with equimolar concentrations of AcuA and AcsA (50-100 μM). The calculated oligomeric states are indicated. All combinations show a stable complex formation as no free AcuA protein can be detected. Source data are provided as Source Data file.

b AcsA from *Chloroflexota bacterium* elutes as apparent monomer from an calibrated analytical Superdex 200 10/300 GL column. *B. subtilis* AcsA was analysed to confirm the dimeric oligomeric state. Source data are provided as Source Data file.

c AlphaFold2 structure of noncomplexed *B. subtilis* AcuA and AcuA in complex with AcsA has a highly similar structure to *E. sibiricum* AcuA (PDB: 2Q04 [https://doi.org/10.2210/pdb2Q04/pdb]) with an overall r.m.s.d. value of 0.7 Å. AlphaFill analyses with AcuA showed that AcuA might coordinate a Ca^{2+} ion with E97, E135 and H139[2]. This is also supported by superposition of the AlphaFold2 structural model of *B. subtilis* AcuA with AcuA from *E. sibiricum*. Notably, H138 of *E. sibiricum* AcuA (superscript E: from *E. sibiricum* AcuA; superscript A: from *B. subtilis* AcuA of *B. subtilis*) is in a different rotamer compared to the homologous H139 of *B. subtilis* AcuA (closeup, left panel). This shows that it might be able to alter its conformation, which is not predicted by AlphaFold2. The position of the positively-charged ion can be replaced by the positively-charged amino group of the K549 side chain of *B. subtilis* AcsA (closeup, right panel).

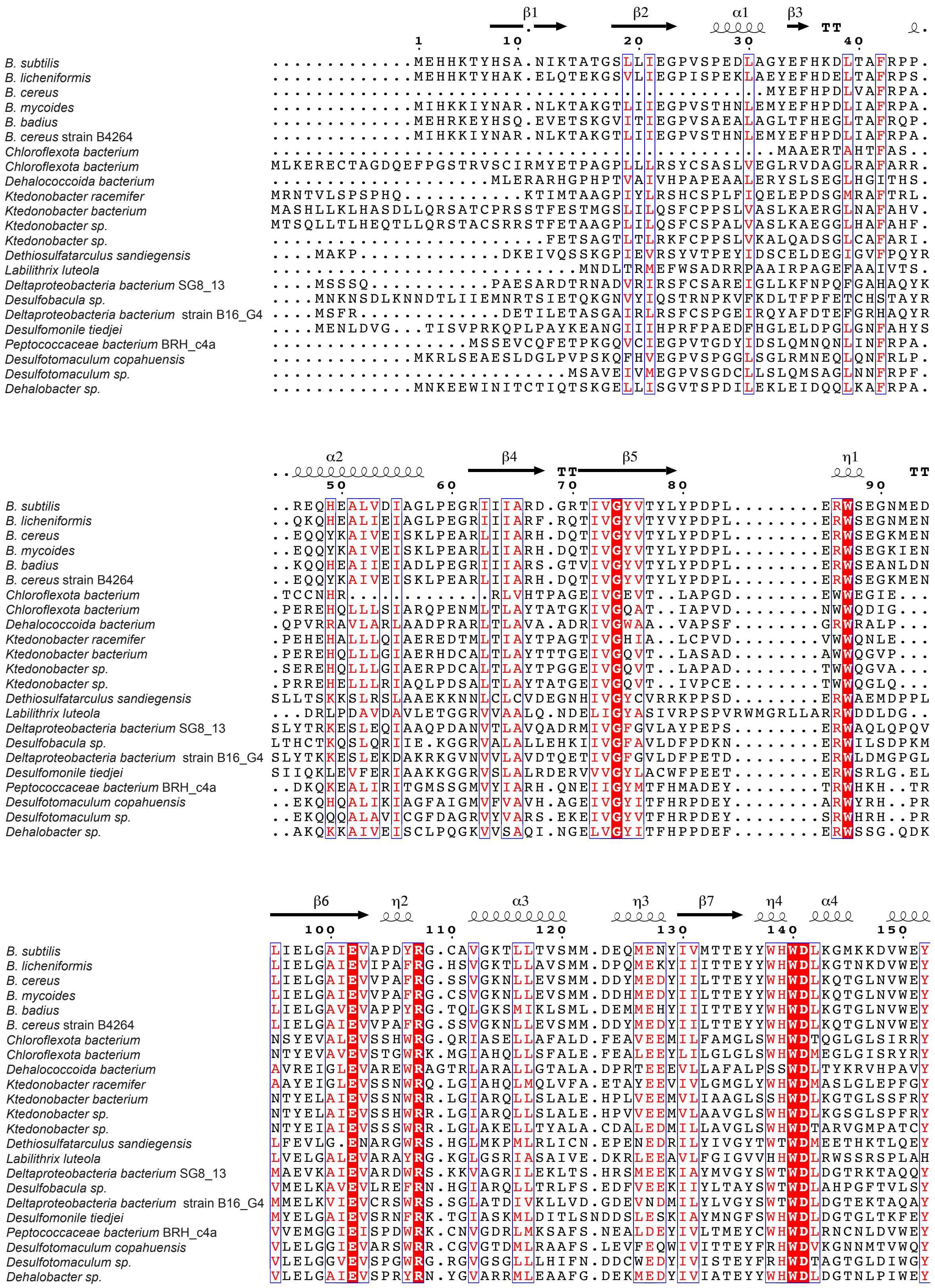
β1
β2
α1
β3
α2
β4
β5
η1
β6
η2
α3
η3
β7
η4
α4
B. subtilis
B. licheniformis
B. cereus
B. mycoides
B. badius
B. cereus strain B4264
Chloroflexota bacterium
Chloroflexota bacterium
Dehalococcoida bacterium
Ktedonobacter racemifer
Ktedonobacter bacterium
Ktedonobacter sp.
Ktedonobacter sp.
Dethiosulfatarculus sandiegensis
Labilithrix luteola
Deltaproteobacteria bacterium SG8_13
Desulfobacula sp.
Deltaproteobacteria bacterium strain B16_G4
Desulfomonile tiedjei
Peptococcaceae bacterium BRH_c4a
Desulfotomaculum copahuensis
Desulfotomaculum sp.
Dehalobacter sp.

	α5 η5 β8 α6 TT β9 TT α7 η6
	160 170 180 190 200 210
B. subtilis	RKIMEKMMNAGGLVWFATDEPEISSHPANCLMARIGKNVSQESIEQFDRLRFYHRYMY..
B. licheniformis	RKMMEKMMNAGGLVWFATDDPEISSHPANCLMARIGKEVSQESIERFDRLRFHNRFMY..
B. cereus	RKVMEKMMNAGGLQWMATDDPEICSHPANCLMVRIGKRVDTDSIQAFDRLRFHNRFMY..
B. mycoides	RKVMEKMMNAGGLQWMATDDPEICSHPANCLMVRIGKRVDTDSIQAFDRLRFHNRFMY..
B. badius	RKIMEKMMNAGGLVWYATDDPEISSHPANCLMVRIGKYVDQAAIERFDQVRFMNRFMY..
B. cereus strain B4264	RKVMEKMMNAGGLQWMATDDPEICSHPANCLMVRIGKRVDTDSIQAFDRLRFHNRFMY..
Chloroflexota bacterium	RVMLSKLFAEQGFVEYPTTEPNIGMDPGNILVARIGKRVDQQVVNRFLHRLLHSPQDGIN
Chloroflexota bacterium	REMIAQLFAAHGFAEYLTSEPNIRMDPANILLARLGSRLEHESMNRFFQRLLQSDT...L
Dehalococcoida bacterium	RRMIAMGLRRYGFVPLATDDPEVCSVPEAALFLRAGARVSAAVLDAVLAARLRTAVRPSQ
Ktedonobacter racemifer	REMLERFCAAYGFTEYLTSEPNIRMHPANIFLARLGKNAKQALVKQFFDGLLRSDT...L
Ktedonobacter bacterium	RSMLAHSLDLYHFFEFMTDEPNIAMDPANILLVRIGQRVKLEVVEKLFQCMRFF......
Ktedonobacter sp.	RSLLAHILEPYHFFECLTDEPNVGMDPANILLVRIGKRVEQEVMDQLFQRMRFF......
Ktedonobacter sp.	RGLIAGIFEPHGFSEYQTAEPNINEDPANILLVRIGNRVEQQAIGQFINRLLGFPD...T
Dethiosulfatarculus sandiegensis	RDTIIHLLTPYGFRQYPTNEPNVSLRAENLFMARLGENIERPVKRNFTNLLFGIRED...
Labilithrix luteola	RRVLSATVRHAGMSSWPTDDPEITMHPANALFARVGPRVAPSLREAFEGKLQGRNAC...
Deltaproteobacteria bacterium SG8_13	RQMLIRLFEPCGFIEYQTNEPNICLKPENLFMGRVGQAVDEQTIQAFKWLRFGIDP....
Desulfobacula sp.	RDMLVSLYTPSGFLEFQTNEPNICLKPENMFMVRMGKKIPQKIQDEFKLLRFGILA....
Deltaproteobacteria bacterium strain B16_G4	RKMLINLFEPHGFEELQTNEPNICLKPENIFMARIGDHISDDVRTQFKWLRFGVTP....
Desulfomonile tiedjei	RKLMLNLLKNYGFRDYYTNEPNVSLREENLFMARIGSEVSEQDRKRFSHLRFGIYD....
Peptococcaceae bacterium BRH_c4a	QKMLTKLFARVGLNKTATDDPDIIEHPANVLMARIGKKVSREDIMKFESMRFLDKPGAG.
Desulfotomaculum copahuensis	RELLTKLFGRVGFVERATSDPDIIEHPANVLMARCGSRVSWDDMMLFEQLLMEKVRWKR.
Desulfotomaculum sp.	RKMLDGFFGRAGFMPVSTNDPDILEHPANVLMARVGSGVGWDDVMDFEEIATGRI.....
Dehalobacter sp.	REMMRHLMTYTDLLIKDTDDEEITSHPANMLMVRYGKNITKKMIYNFDRLLFLNGK....

B. subtilis	....
B. licheniformis	....
B. cereus	....
B. mycoides	....
B. badius	....
B. cereus strain B4264	....
Chloroflexota bacterium	DGV......
Chloroflexota bacterium	PGL......
Dehalococcoida bacterium	RIHLPVTAA
Ktedonobacter racemifer	PGL......
Ktedonobacter bacterium	....
Ktedonobacter sp.	....
Ktedonobacter sp.	PTFP.....
Dethiosulfatarculus sandiegensis	....
Labilithrix luteola	....
Deltaproteobacteria bacterium SG8_13	....
Desulfobacula sp.	....
Deltaproteobacteria bacterium strain B16_G4	....
Desulfomonile tiedjei	....
Peptococcaceae bacterium BRH_c4a	....
Desulfotomaculum copahuensis	....
Desulfotomaculum sp.	....
Dehalobacter sp.	....

Supplementary Figure 9: Sequence alignment of selected AcuA enzymes.

The alignment was conducted to uncover conserved regions to *B. subtilis* AcuA (uniport: P39065). For AcuA we observed that in bacterial species of the genera *Bacillus*, *Chlostridium*, *Chloroflexota* and *Salmonella* the catalytic glutamate (*B. subtilis* AcuA: E102) and the W140 (*B. subtilis*: 139-WHW-140) is totally conserved. The *B. subtilis* AcuA E135 is conserved only in some sequences sometimes replaced by an alanine or glycine maybe indicating an improved Pta activity compared to *B. subtilis* AcsA•AcuA (P39065: *B. subtilis* AcuA; Q65G33: *Bacillus licheniformis* AcuA; A0A068NCR1: *Bacillus cereus* AcuA; A0A084IVY2: *Bacillus mycoides* AcuA; A0A0C2Y8F1: *Bacillus badius* AcuA; J3ZWF0: *Bacilllus cereus* strain B4264 AcuA; A0A535FXN8: *Chloroflexota bacterium* AcuA; A0A536GMG9: *Chloroflexota bacterium* GNAT family N-acetyltransferase; A0A7V1VYL1: *Dehalococcoidia bacterium* GNAT family N-acetyltransferase; D6U4Q1: *Ktedonobacter racemifer* AcuA; A0A838EVD4: *Ktedonobacter bacterium* GNAT family N-acetyltransferase; A0A1Q6ZW01: *Ktedonobacter sp.* 13_2_20CM_2_56_8 N-acetyltransferase domain-containing protein; A0A349M2Y3: *Ktedonobacter sp.* N-acetyltransferase domain-containing protein; A0A0D2GLU4: *Dethiosulfatarculus sandiegensis* N-acetyltransferase domain-containing protein; A0A0K1Q151: *Labilithrix luteola* AcuA; A0A0S7YQC5: *Deltaproteobacteria bacterium* SG8_13 N-acetyltransferase domain-containing protein; A0A1F9NWB7: *Desulfobacula sp.* RIFOXYA12_FULL_46_16 N-acetyltransferase domain-containing protein; A0A509L2H4: *Deltaproteobacteria bacterium* strain B16_G4 N-acetyltransferase; I4CA61: *Desulfomonile tiedjei* N-acetyltransferase domain-containing protein; A0A0F2N3N8: *Peptococcaceae bacterium* BRH_c4a acetoin dehydrogenase; A0A1B7LHY8: *Desulfotomaculum copahuensis* N-acetyltransferase domain-containing protein; A0A358QRQ7: *Desulfotomaculum sp.* N-acetyltransferase; T0IDI1: *Dehalobacter sp.* UNSWDHB AcuA). The secondary structure elements are given on the top for *B. subtilis* AcuA. The sequence alignments were conducted with ClustalW and ESPript 3.0 was used to render sequence similarities and information on secondary structure elements derived from the AlphaFold2 structure predictions.

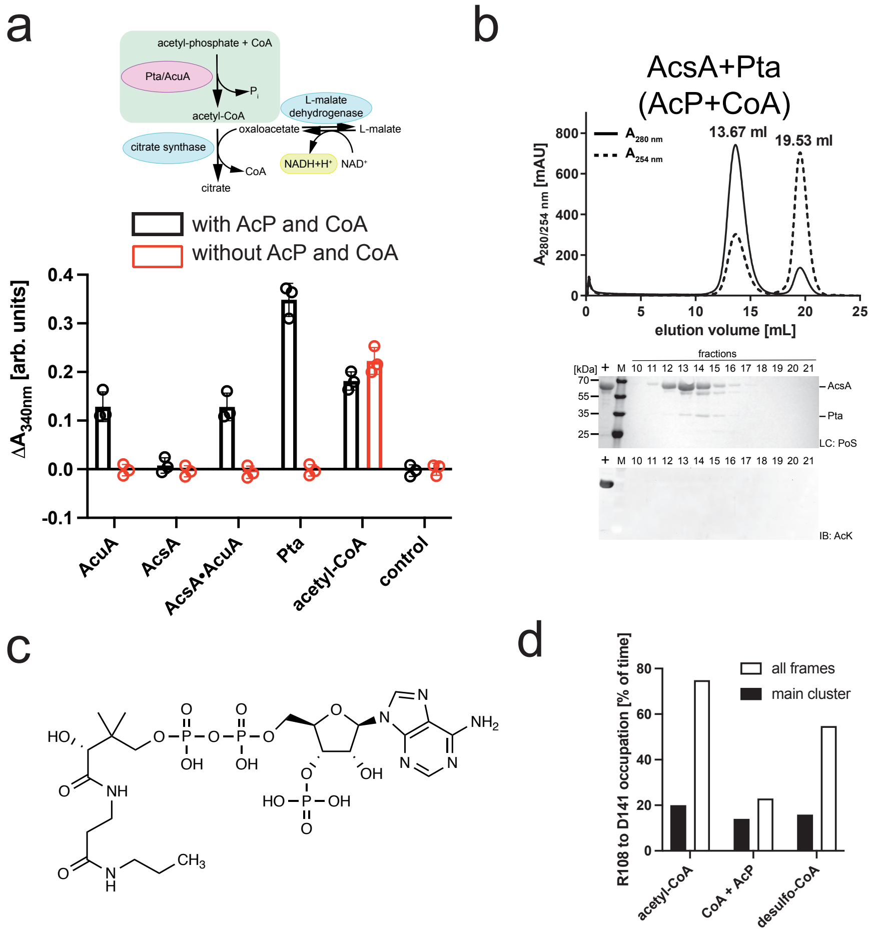

Supplementary Figure 10: The complex of AcsA•AcuA is not affected by CoA and AcP alone.

a AcsA•AcuA and AcuA have an intrinsic Pta activty. To show if AcuA or the AcsA•AcuA complex has a Pta activity, a coupled enzymatic assay was performed to detect the generated acetyl-CoA (upper panel). The purified *B. subtilis* Pta is active in generating acetyl-CoA from AcP and CoA. Notably, AcuA alone and the AcsA•AcuA complex resulted in similar levels of acetyl-CoA suggesting that AcuA is sufficent as phosphotransacetylase (Pta) to catalyse the formation of acetyl-CoA from AcP and CoA. arb. units: arbitrary units. The control is the reaction without acetyl-CoA and lacking Pta, AcsA and/or AcuA. The experiment was performed in three biologically independent replicates (n=3). Shown are means ±SD. Source data are provided as Source Data file.

b The SEC run of AcsA in presence of *B. subtilis* phosphotransacetylase (Pta) was conducted as control to exclude the possibility that a potential Pta activity present as contamination in the protein preparations results in formation of acetyl-CoA that is used to acetylate AcsA. AcsA elutes as dimer (13.67 ml) and is not acetylated shown by immunoblotting using an anti-AcK-AB (IB: AcK). PoS: Ponceaut S-red staining was done as loading control. The A_{254} nm peak at 19.53 ml corresponds to CoA/acetyl-CoA. The lane labelled with + shows the acetylated *B. subtilis* AcsA loaded as technical control for the immunoblot, the lane labelled with M represents the protein molecular weight marker. Source data are provided as Source Data file and in Supplementary Fig. 18.

c Chemical structure of desulfo-CoA. In desulfo-CoA the thiol group of CoA is replaced by a methyl-group. This reduces the reactivity switching-off its capacity to act as nucleophile upon deprotonation.

d MD simulations reveal the contact time between R108 of AcuA and D141 of AcuA in the models with acetyl-CoA, CoA plus AcP and desulfo-CoA. The interaction between R108 and D141 is most frequently observed in AcuA of the AcsA•AcuA complex in complex with acetyl-CoA. In presence of CoA and AcP the frequency is strongly reduced suggesting a competition between the negatively-charged phosphoryl-group of AcP and the negatively-charged D141 for binding to R108. In absence of AcP, in the desulfo-CoA containing model, the interaction frequency between R108 and D141 is increased but does not reach the level obtained for the model with acetyl-CoA supporting the hypothesis that AcP competes with D141 for binding to R108. Source data are provided as Source Data file.

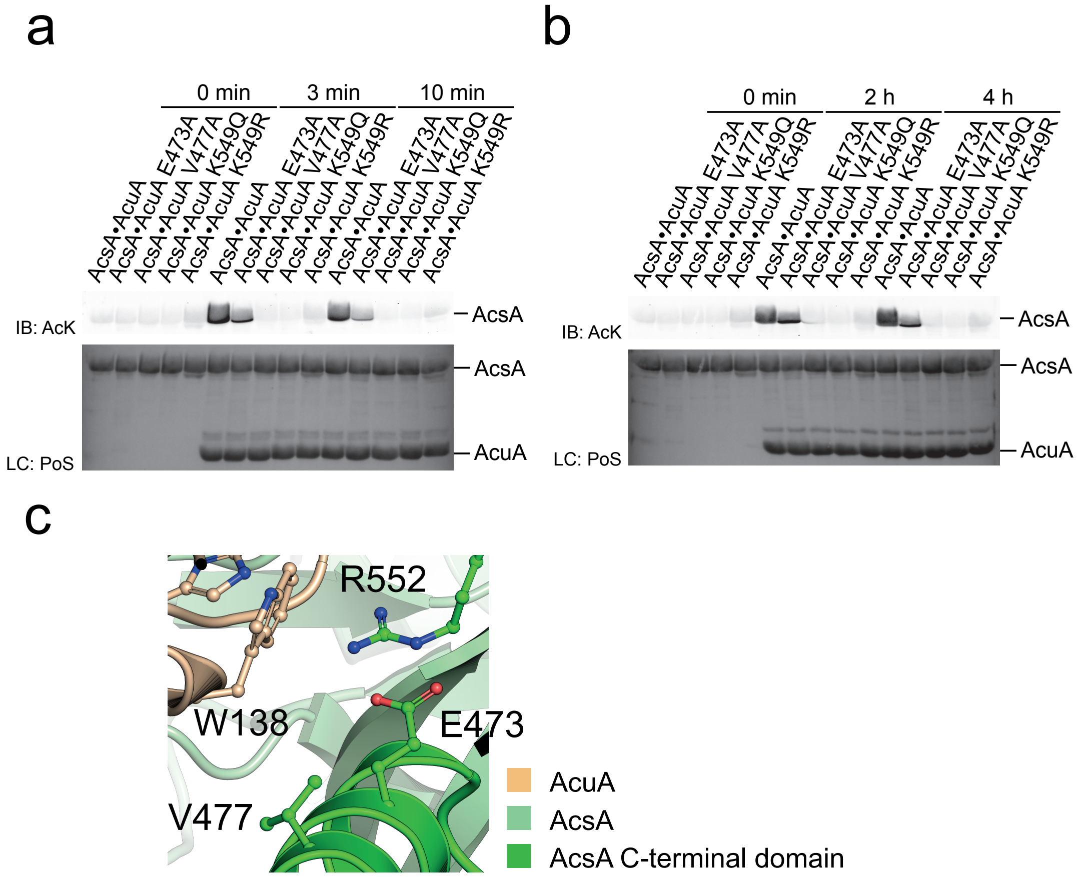

Supplementary Figure 11: AcsA mutants E473A and V477A impair AcT activity of AcsA•AcuA, K549 of AcsA is the acetyl-group acceptor for AcT and Pta activity of AcsA•AcuA.

a AcT activity of AcuA on AcsA is impaired by AcsA E473A and V477A. For AcsA E473A and V477A a reduced acetylation was observed indicating that these mutations affect the AcT activity of AcsA•AcuA. E473 is in interaction distance to R552, which indirectly forms an electrostatic interaction with the aromatic side chain of W138 of AcuA (see panel C). V477 of AcsA is located on the same α-helix as E473 most likely playing a structural role (see panel C). Both mutations reduce the affinity of AcsA and AcuA as shown by analytical SEC (Supp. Fig. S5). As shown before, AcsA K549R and K549Q are not acetylated confirming that K549 is the major acetyl-group acceptor site for acetylation by AcuA. AcsA (10 μM) was incubated with/without AcuA (10 μM) and acetyl-CoA (200 μM) for 0 min, 3 min or 10 min, as indicated. AcsA acetylation was assessed by immunoblotting using an anti-AcK-AB (IB: AcK). Loading control was done by Ponceau S-red staining (LC: PoS). Source data are provided in Supplementary Fig. 19.

b Phosphotransacetylase activity of AcuA on AcsA is not affected by AcsA E473A and V477A. AcsA V477A and E473A behave as observed in the AcT assay, i.e. they do not discriminate between Pta activity and AcT activity, suggesting that these mutations affect the AcT activity used as readout for Pta activity. As explained for the AcT activity K549 is the major acetyl-group acceptor site for acetylation by AcuA. AcsA (10 μM) was incubated with/without AcuA (10 μM), AcP (2 mM) and CoA (2 mM) for 0 h, 2 h or 4 h, as indicated. AcsA acetylation was assessed by immunoblotting using an anti-AcK-AB (IB: AcK). Loading control was done by Ponceau S-red staining (LC: PoS). Source data are provided in Supplementary Fig. 20.

c Orientation of E473 and V477 of AcsA in the AcsA•AcuA structure predicted by AlphaFold2. E473 and V477 are both located in the C-terminal domain of AcsA. E473 is in direct intramolecular contact to R552 of AcsA, which contacts W138 of AcuA forming an electrostatic interaction of the positively-charged guanidino group of R552 and the π-electron system of the aromatic side chain of W138. V477 is located in the same α-helix as E477 playing a structural role. E473 of AcsA is totally conserved and with V477 of AcsA in direct vicinity to core motif A8, containing D457, the hinge for the conformational change, R466 essential for binding of the AMP-ribose and G464 for lining the CoA binding tunnel in the thioester-forming conformation (Supplementary Fig. 1; Supplementary Fig. 13). Next to impairing the affinity of AcsA and AcuA, these mutations might therefore also have a catalytic role on AcsA activity.

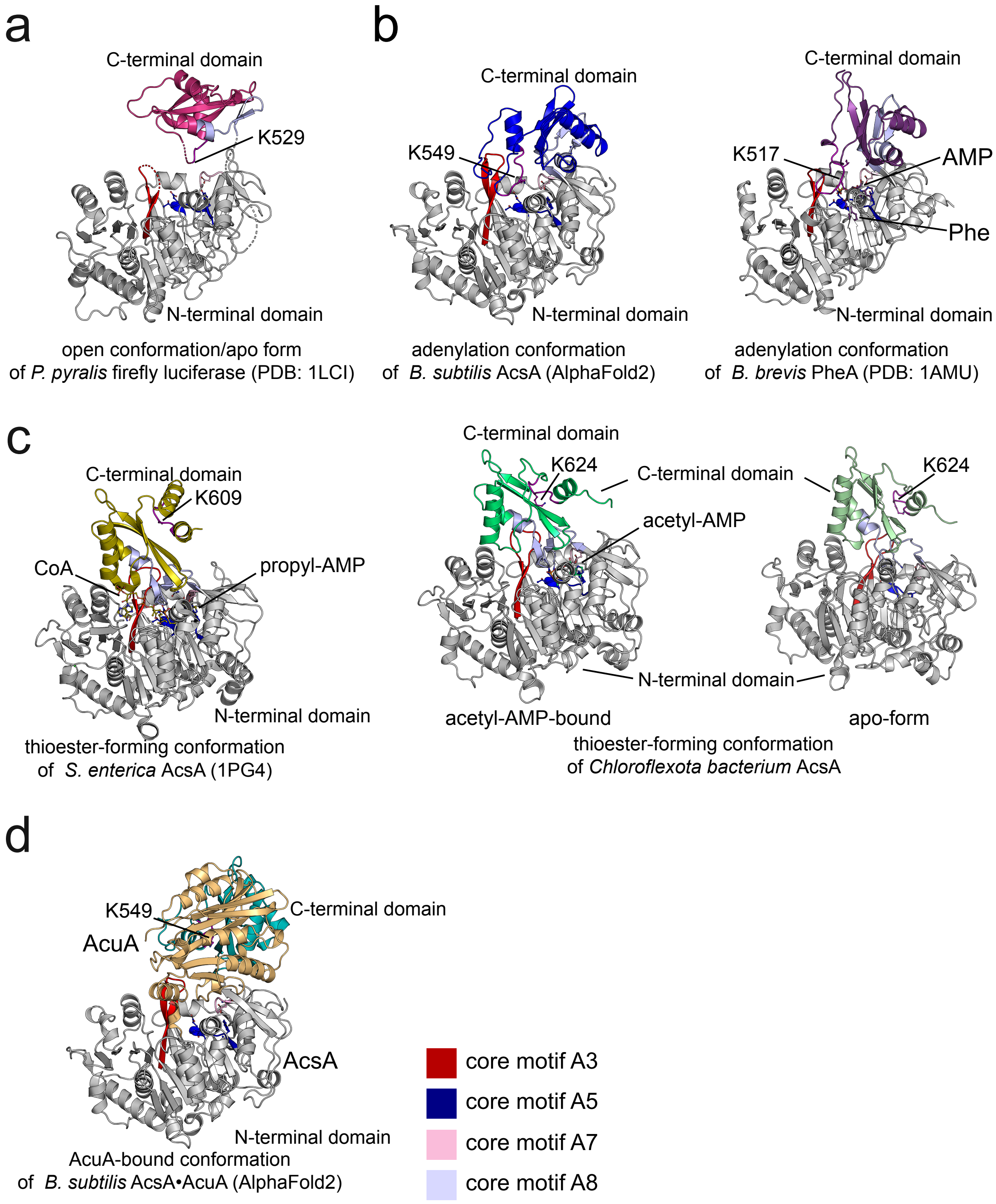

Supplementary Figure 12: Structural comparison of the structures of *Chloroflexota bacterium* AcsA in the apo form (PDB: 8RPK [https://doi.org/10.2210/pdb8RPK/pdb]) and acetyl-AMP bound form (PDB: 8RPL [[https://doi.org/10.2210/pdb8RPL/pdb]) to known structures of enzymes of the ANL-superfamily.

a Structure of *Photinus pyralis* firefly luciferase (PDB: 1LCI [https://doi.org/10.2210/pdb1LCI/pdb]) in its unliganded apo state. The C-terminal domain (pink) is detached from the N-terminal domain (grey) the connecting linker is not build suggesting a high degree of flexibility concerning its orientation to the N-terminal domain. The core motifs are color coded as indicated.

b Structures of enzymes of the ANL-superfamily in the conformation to catalyse the first half-reaction (adenylation conformation). The AlphaFold2 model of *B. subtilis* AcsA and the enzyme PheA (PDB: 1AMU [https://doi.org/10.2210/pdb1AMU/pdb]) from *Brevibacillus brevis* in complex with AMP and the substrate phenylalanine adapt the adenylation conformation. The conserved lysine of core motif A10 (*B. subtilis* AcsA: K549; *B. brevis* PheA: K525) interacts with the ribose of the AMP. Binding of the β,γ-phosphates of ATP is achieved by motif A3 (phosphate binding loop). The core motifs are color coded as indicated.

c Structures of AcsA from *Salmonella enterica* in complex with CoA and propyl-AMP (PDB: 1PG4 [https://doi.org/10.2210/pdb1PG4/pdb]) and *Chloroflexota bacterium* (apo form (PDB: 8RPK [https://doi.org/10.2210/pdb8RPK/pdb]) and acetyl-AMP-bound form (PDB: 8RPL [https://doi.org/10.2210/pdb8RPL/pdb])) are shown in the conformation capable to catalyze the second half-reaction (thioester-forming conformation). Although suggested for *S. enterica* Acs CoA binding mediating the stabilization of the thioester-forming conformation, the structure of *Chloroflexota bacterium* AcsA in the apo form and in complex with acetyl-AMP presented here adapts an almost identical conformation. This conformation is stabilized by residues of core motif A5 and A8. In the apo form, the C-terminal is marginally shifted compared to the structure in complex with acetyl-AMP. The core motifs are color coded as indicated.

d AlphaFold2 model of the AcsA•AcuA structure from *B. subtilis*. Binding of AcuA to AcsA stabilizes AcsA in a fourth conformation, in which K549 of core motif A10 is bound in the AcuA active site. The C-terminal domain adapts a conformation that is incompatible to catalyse both half-reactions. The core motifs are color coded as indicated[3].

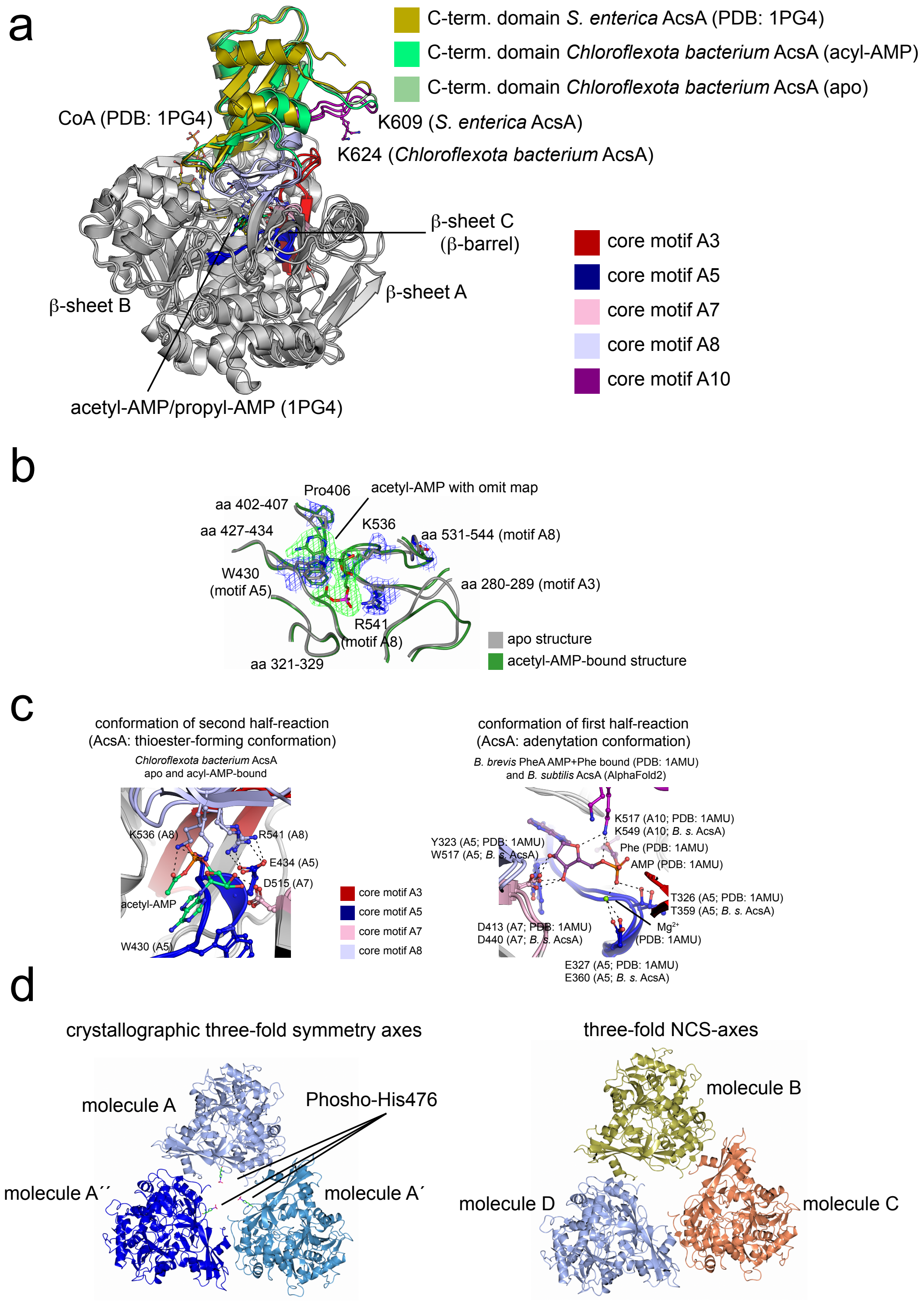

Supplementary Figure 13: Crystal structure of *Chloroflexota bacterium* AcsA in complex with acetyl-AMP (PDB: 8RPL [https://doi.org/10.2210/pdb8RPL/pdb]) and in its unliganded apo form (PDB: 8RPK [https://doi.org/10.2210/pdb8RPK/pdb]**).**

a Superposition of *Chloroflexota bacterium* AcsA in complex with acetyl-AMP, the unliganded apo form and *S. enterica* AcsA (PDB: 1PG4 [https://doi.org/10.2210/pdb1PG4/pdb]) in complex with CoA and propyl-AMP. All structures represent the thioester-forming conformation and are highly similar with r.m.s.d values of 0.674 Å obtained for 1PG4 [https://doi.org/10.2210/pdb1PG4/pdb] to the apo form and of 0.645 Å to the acetyl-AMP-bound structure of *Chloroflexota bacterium* determined here. The conserved lysine (K524 for *Chloroflexota bacterium* and K609 for *S. enterica* Acs) is exposed and accessible for enzymatic acetylation or deacetylation. The *Chloroflexota bacterium* structures show marginal variations in the orientation of the C-terminal domain to the N-terminal domain. The N-terminal domains are shown in grey, the C-terminal domains are color coded as indicated.

b Electron density for the AcsA *Chloroflexota bacterium* structure with acetyl-AMP (PDB: 8RPL [https://doi.org/10.2210/pdb8RPL/pdb]) and superposition with the apo structure (PDB: 8RPK [https://doi.org/10.2210/pdb8RPK/pdb]). The structure of *Chloroflexota bacterium* AcsA with acetyl-AMP is shown with dark green carbons, the apo structure with grey carbons. Notice rearrangement of side chains on the right side of the binding site and concurrent main chain shifts, local in the N-terminal domain, but leading to 1-2 Å shifts throughout the C-terminal domain. A distinct main chain rearrangement is seen in a peptide flip between residues 532 and 533 (upper right). Omit density for acetyl AMP is shown in green at 5 σ contour level, $2F_o$-F_c density of nearby residues is shown in blue at 1.5 σ contour level.

c Closeup of the interactions observed in the conformation of the first and second half-reactions. Left panel: The *Chloroflexota bacterium* AcsA is an example for an AcsA in the thioester-forming conformation. The structure was solved in complex with the product of the adenylation reaction, i.e. acetyl-AMP (PDB: 8RPL [https://doi.org/10.2210/pdb8RPL/pdb]). The conformation is stabilized by residues of core motifs A3 and A10 and in state competent to bind CoA. R541 of motif A8 forms a salt bridge to E434 of core motif A3. This R541 is solvent exposed in the adenylation conformation. W430 of motif A5 stacks against the adenine base of the acetyl-AMP. K536 of motif A8 forms an interaction with the bound acetyl-AMP by contacting the carbonyl oxygen of the acetyl-group, C-5' oxygen of the ribose and the C-5'-phosphate. Right panel: *B. brevis* PheA PheA (PDB: 1AMU [https://doi.org/10.2210/pdb1AMU/pdb]) was solved in complex with AMP and phenylalanine, i.e. the substrates of the first half-reaction representing the adenylation conformation. The PheA structure was superimposed with the AlphaFold2 structure of *B. subtilis* AcsA. This conformation is primarily stabilized by residues of core motif A3 (phosphate binding motif), that contacts the β/γ-phosphates of the ATP substrate (not visible here) and by residues from core motif A10. K549/K524 contact the C-4' and C-5' oxygens of the ribose. The core motifs are color coded as indicated.

d The apo form (PDB: 8RPK [https://doi.org/10.2210/pdb8RPK/pdb]) and acetyl-AMP-form (PDB: 8RPL [https://doi.org/10.2210/pdb8RPL/pdb]) of *Chloroflexota bacterium* AcsA contains a phospho-histidine. The *Chloroflexota bacterium* AcsA structure contains four molecules per asymmetric unit. It forms a monomer in solution (Supplementary Fig. 8b). However, we observed that the three-fold crystallographic symmetry axis and a non-crystallographic three-fold symmetry axis both orient symmetrical trimers in the same way such that the phospho-His476 of each monomer is facing the symmetry axis. Future studies are needed to identify how His476 is phosphorylated and whether this phosphorylation is of any physiological significance such as formation of a trimer if a positively-charged ion or molecule is placed in the center.

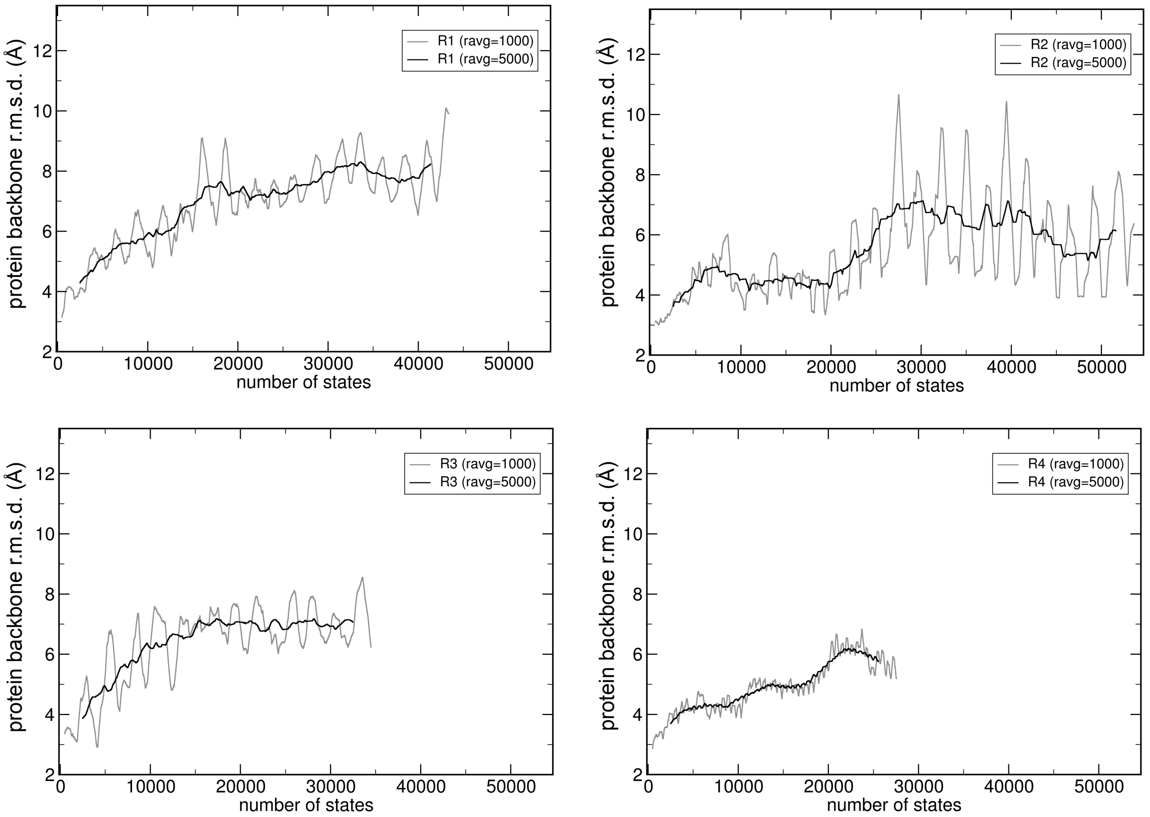

Supplementary Fig. 14: Running average (window length of 1000 and 5000 states) of protein backbone RMSD of AcuA + AcsA complexes duringTIGER2h$_{PE}$ replica-exchange MDS to access convergence of ensembles. R1-R4 refer to the individual simulation runs as reported in Supplementary Table 2.

SDS-PAGE, Coomassie-brilliant blue (CBB)

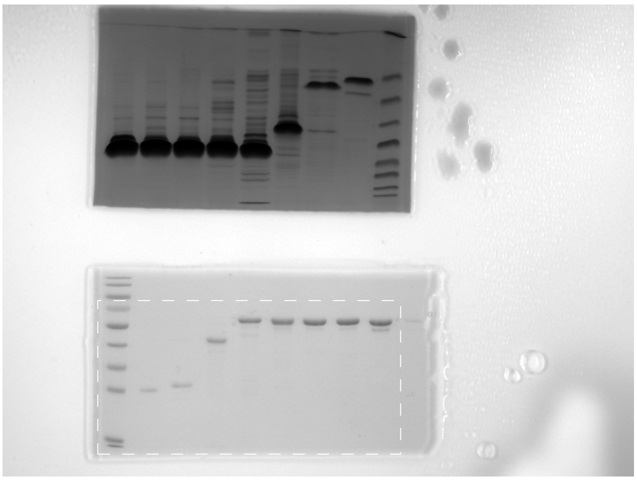

SDS-PAGE, Coomassie-brilliant blue (CBB)

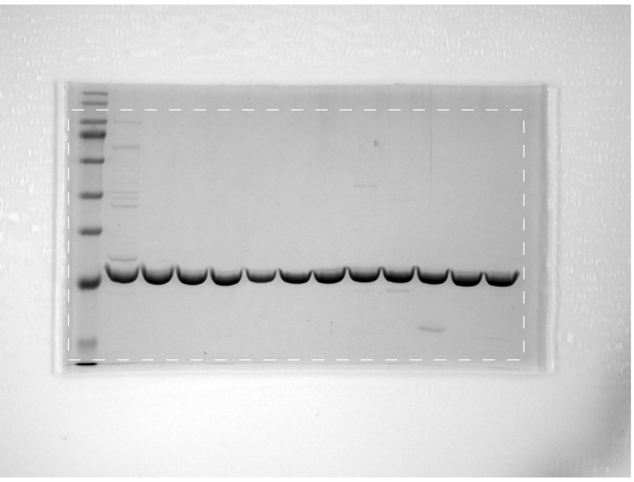

Supplementary Figure 15: Source Data Supplementary Fig. 2a.

immunoblotting

CoA AcP

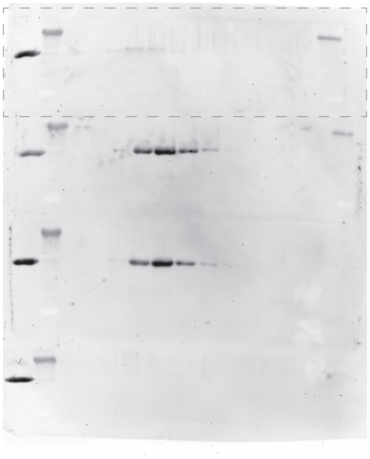

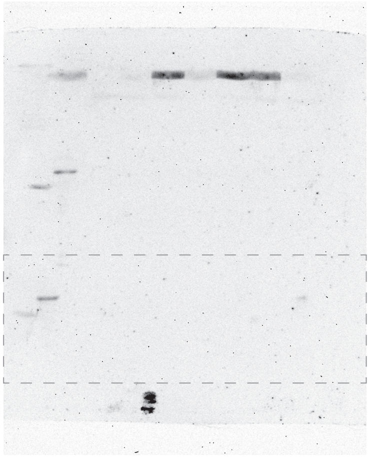

loading control, Ponceau S-red staining

CoA AcP

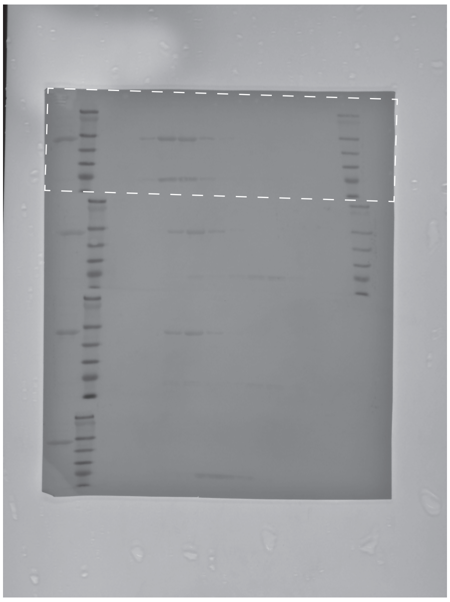

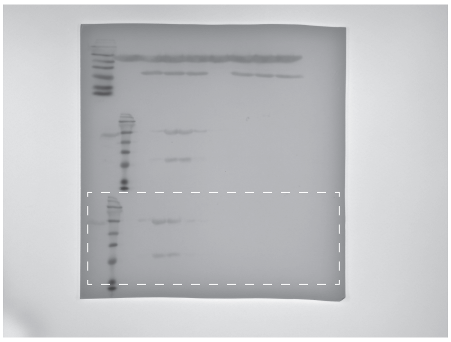

Supplementary Figure 16: Source Data Supplementary Fig. 4c.

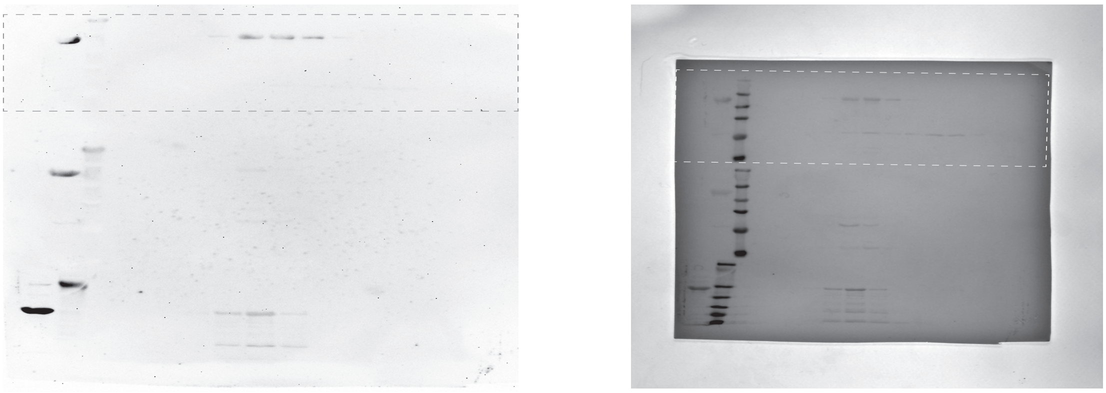

Supplementary Figure 17: Source Data Supplementary Fig. 5a.

SEC, immunoblotting SEC, loading control, Ponceau S-red staining

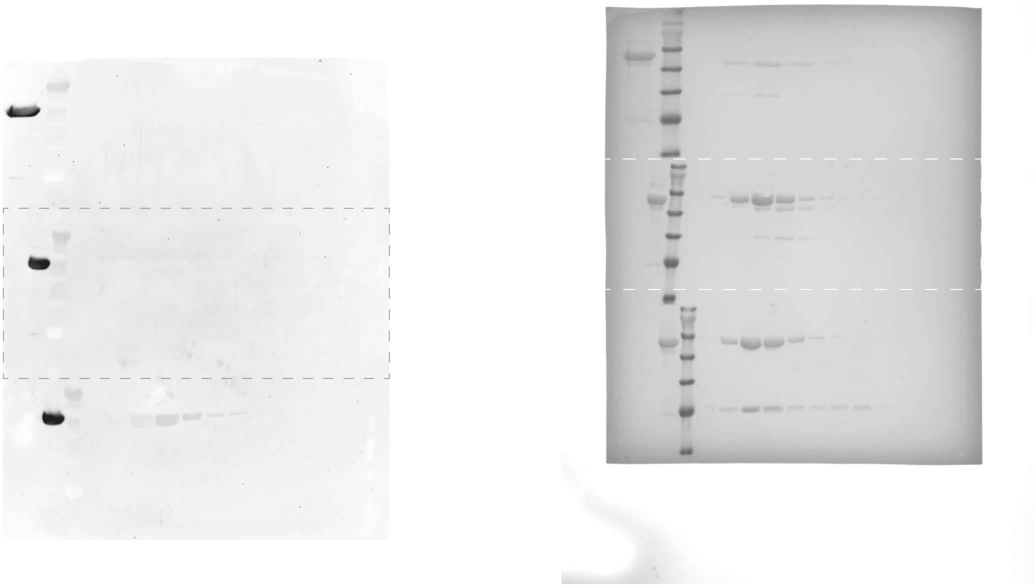

Supplementary Figure 18: Source Data Supplementary Fig. 10b.

immunoblotting loading control, Ponceau S-red

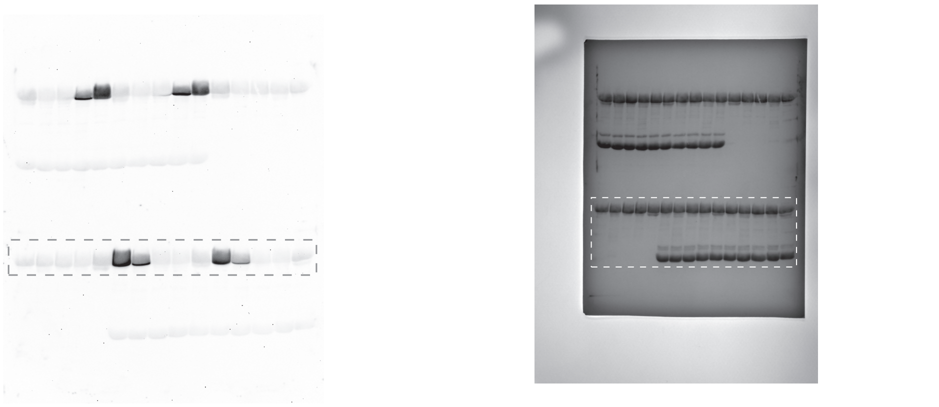

Supplementary Figure 19: Source Data Supplementary Fig. 11a.

immunoblotting loading control, Ponceau S-red

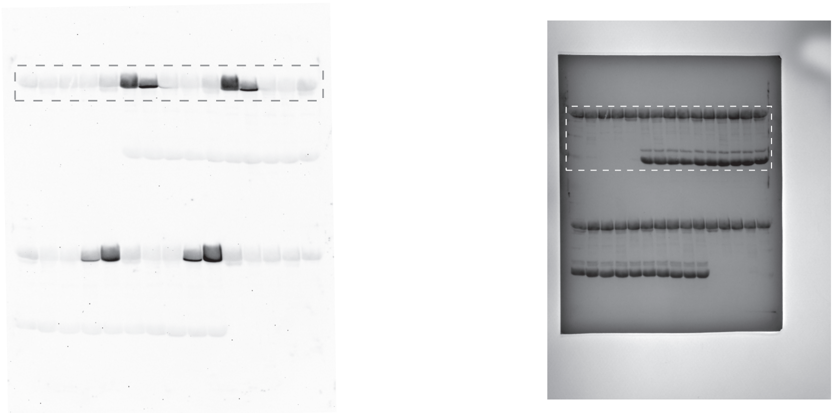

Supplementary Figure 20: Source Data Supplementary Fig. 11b.

Supplementary Table 1: Data collection and refinement statistics (molecular replacement) for the structures of *Chloroflexota bacterium* AcsA in complex with acetyl-AMP or in the apo form.

	AcsA•AMP[1*] (PDB: 8RPL [https://doi.org/10.2210/pdb8RPL/pdb])	apo AcsA[2*] (PDB: 8RPK [https://doi.org/10.2210/pdb8RPK/pdb])
**Data collection**		
Space group	H 3 2	H 3 2
Cell dimensions		
a, *b*, *c* (Å)	161.8, 161.8, 816.7	162.2, 162.2, 817.7
α, β, γ (°)	90, 90, 120	90, 90, 120
Resolution (Å)	27.2 (2.37)[3*]	27.2 (3.04)
R_{merge}	0.12 (2.90)	0.17 (1.86)
$I / \sigma I$	16.9 (1.1)	12.5 (1.7)
Completeness (%)	100 (100)	95.1 (56.4)
Redundancy	20.4 (21.5)	18.5 (18.0)
**Refinement**		
Resolution (Å)	2.37	3.04
No. reflections	167482	66860
R_{work} / R_{free}	0.161 / 0.202	0.164 / 0.210
No. atoms		
Protein	20138	20018
Ligand/ion	224	61
Water	181	36
B-factors		
Protein (main chain/side chain)	73.4/80.3	76.9/81.9
Ligand (acetyl-AMP)	63.7	-
…Ligand/ion	125.6	120.9
Water	65.4	66.5
R.m.s. deviations		
Bond lengths (Å)	0.007	0.008
Bond angles (°)	1.69	1.90

[1*]/[2*]: for each structure one xtal was used.
[3*]: values in parentheses are for highest-resolution shell.

Supplementary Table 2: Overview of simulated systems, denoting system content, total simulation time across all replicas without quenching phases and total number of cycles, average temperature changes during replica swaps for TIGER2h-PE algorithm and average swap probability. The number of cycles is also the number of structures generated.

Run	*System content*	*T [μs] (cycles)*	*ΔT/X [K]*	*P(X) [%]*
R1	AcuA•AcsA, Acetyl-CoA	5.6 (43871)	8.7	22.0
R2	AcuA•AcsA, Acetyl-CoA, deprotonated K549 in AcuA	6.9 (54109)	10.2	24.3
R3	AcuA•AcsA, CoA, AcP	4.5 (34998)	9.8	24.0
R4	AcuA•AcsA, Desulfo-CoA	3.6 (28060)	8.7	22.0

Supplementary Table 3: Results of RMSD cluster analysis after application of contact filters. Numbers denote the percentage of frames assigned to the first 5 clusters and the corresponding numbers of frames in braces.

Run	*Main Cluster*	*2nd*	*3rd*	*4th*	*5th*
R2 (Acetyl-CoA)	51.3 (299)	13.6 (79)	8.9 (50)	6.2 (36)	4.3 (27)
R3 (CoA)	78.6 (5511)	11.0 (700)	2.51 (170)	1.4 (142)	1.3 (84)
R3 (AcP)	37.1 (73)	12.2 (24)	11.2 (22)	9.1 (18)	7.1 (14)
R4 (desulfo-CoA)	53.5 (1709)	17.2 (454)	7.6 (235)	7.5 (215)	2.7 (86)

References:

1. The PyMOL Molecular Graphics System, Version 3.0, Schrödinger, LLC.
2. Hekkelman, M.L., de Vries, I., Joosten, R.P. & Perrakis, A. AlphaFill: enriching AlphaFold models with ligands and cofactors. *Nat Methods* **20**, 205-213 (2023).
3. Jumper, J. et al. Highly accurate protein structure prediction with AlphaFold. *Nature* **596**, 583-589 (2021).
