# Supplementary material for: Acetyl-CoA synthetase activity is enzymatically regulated by lysine acetylation using acetyl-CoA or acetyl-phosphate as donor molecule
Source: Nat Commun. 2024 Jul 17;15:6002. doi: 10.1038/s41467-024-49952-0 (PMC11255334; doi:10.1038/s41467-024-49952-0)
Supplement: Supplementary file 3 — Description of Additional Supplementary Files [file 41467_2024_49952_MOESM3_ESM.pdf]

Description of Additional Supplementary Files

File Name: Supplementary Data 1

Description: AlphaFold2 structure prediction of the structures of Bacillus subtilis AcsA, AcuA, complexes and mutants thereof. AlphaFold2 version 2.2.4 was used to predict the structures. As output files, PDB coordinate files were generated. For the monomers, average pLDDT (predicted local distance difference test)-scores were obtained to judge the quality and confidence of the AlphaFold2 structure predictions. The model with the highest overall pLDDT-score was used for further analyses. To judge the quality and confidence of the AlphaFold2 structure predictions for the multimers the ipTM (interface pTM)+pTM (predicted TM)-scores were calculated. The model with the highest overall ipTM+pTM-score was used for further analyses. The pLDDT-scores and ipTM+pTM scores are provided in the text file. We predicted the following monomer, dimer, complex structures and the mutants thereof: AcsA monomer, AcuA monomer, AcsA dimer, AcsA•AcuA, AcsA K549Q•AcuA, AcsA K549R•AcuA, AcsA E437A•AcuA, AcsA V477A•AcuA, AcsA•AcuA E85A, AcsA•AcuA E85Q, AcsA•AcuA E97A, AcsA•AcuA E97Q, AcsA•AcuA E85Q E97Q, AcsA•AcuA E102A, AcsA•AcuA E102Q, AcsA•AcuA E135A, AcsA•AcuA E135Q, AcsA•AcuA E97Q E135Q, AcsA•AcuA H139A, AcsA•AcuA W140A, AcsA•AcuA W140F.
