# Supplementary material for: Acetyl-CoA synthetase activity is enzymatically regulated by lysine acetylation using acetyl-CoA or acetyl-phosphate as donor molecule
Source: Nat Commun. 2024 Jul 17;15:6002. doi: 10.1038/s41467-024-49952-0 (PMC11255334; doi:10.1038/s41467-024-49952-0)
Supplement: Supplementary file 4 — Supplementary Data 1 [file 41467_2024_49952_MOESM4_ESM.docx]

**Supplementary Data**

**Acetyl-CoA synthetase activity is controlled by lysine acetylation using acetyl-CoA or acetyl-phosphate as donor molecules**

Chuan Qin^1^, Leonie G. Graf^1^, Kilian Striska^1^, Markus Janetzky^1^, Norman Geist^2^, Robin Specht^3^, Sabrina Schulze^1^, Gottfried J. Palm^1^, Britta Girbardt^1^, Babett Dörre^1^, Leona Berndt^1^, Stefan Kemnitz^4^, Mark Doerr^3^, Uwe T. Bornscheuer^3^, Mihaela Delcea^2^, and Michael Lammers^1*^

^1^University of Greifswald, Institute of Biochemistry, Department of Synthetic and Structural Biochemistry, 17489 Greifswald, Germany

^2^University of Greifswald, Institute of Biochemistry, Department of Biophysical Chemistry, 17489 Greifswald, Germany

^3^University of Greifswald, Institute of Biochemistry, Department of Biotechnology & Enzyme Catalysis, 17489 Greifswald, Germany

^4^University of Greifswald, University Computing Center, Department for High Performance Computing, 17489 Greifswald, Germany

^*^correspondence should be addressed to Michael Lammers; Email: michael.lammers@uni-greifswald.de; Tel.: +49-(0)3834-420-4356

**Supplementary Data 1: AlphaFold2 structure prediction of the monomer of *Bacillus subtilis* AcsA.** AlphaFold2 version 2.3.1 was used to predict the structure (Supplementary Fig. 2; Supplementary Fig.3). As output files, this PDB coordinate files was generated.

**Supplementary Data 2: Data quality and confidence score for the AlphaFold2 structure prediction of the monomer of *Bacillus subtilis* AcsA.** To judge the quality and confidence of the AlphaFold2 structure predictions pLDDT (predicted local distance difference test)-scores for the monomers were generated. The model with the highest overall pLDDT score was used for further analyses.

**Supplementary Data 3: AlphaFold2 structure prediction of the monomer of *Bacillus subtilis* AcuA (*Bs*AcuA).** AlphaFold2 version 2.3.1 was used to predict the structure (Supplementary Fig. 2; Supplementary Fig.3). As output files, this PDB coordinate files was generated.

**Supplementary Data 4: Data quality and confidence score for the AlphaFold2 structure prediction of the monomer of *Bacillus subtilis* AcuA (*Bs*AcuA).** To judge the quality and confidence of the AlphaFold2 structure predictions pLDDT (predicted local distance difference test)-scores for the monomers were generated. The model with the highest overall pLDDT score was used for further analyses.

**Supplementary Data 5: AlphaFold2 structure prediction of the monomer of *Bacillus subtilis* AcsA K549Q.** AlphaFold2 version 2.3.1 was used to predict the structure (Supplementary Fig. 2; Supplementary Fig.3). As output files, this PDB coordinate files was generated.

**Supplementary Data 6: Data quality and confidence score for the AlphaFold2 structure prediction of the monomer of *Bacillus subtilis* AcsA K549Q.** To judge the quality and confidence of the AlphaFold2 structure predictions pLDDT (predicted local distance difference test)-scores for the monomers were generated. The model with the highest overall pLDDT score was used for further analyses.

**Supplementary Data 7: AlphaFold2 structure prediction of the monomer of *Bacillus subtilis* AcsA K549R.** AlphaFold2 version 2.3.1 was used to predict the structure (Supplementary Fig. 2; Supplementary Fig.3). As output files, this PDB coordinate files was generated.

**Supplementary Data 8: Data quality and confidence score for the AlphaFold2 structure prediction of the monomer of *Bacillus subtilis* AcsA K549R.** To judge the quality and confidence of the AlphaFold2 structure predictions pLDDT (predicted local distance difference test)-scores for the monomers were generated. The model with the highest overall pLDDT score was used for further analyses.

**Supplementary Data 9: AlphaFold2 structure prediction of the dimer of *Bacillus subtilis* AcsA.** AlphaFold2 version 2.3.1 was used to predict the structure (Supplementary Fig. 2; Supplementary Fig.3). As output files, this PDB coordinate files was generated.

**Supplementary Data 10: Data quality and confidence score for the AlphaFold2 structure prediction of the dimer of *Bacillus subtilis* AcsA.** To judge the quality and confidence of the AlphaFold2 structure predictions for the multimers the iptm (interface ptm)+ptm (predicted TM)-scores were calculated. The model with the highest overall iptm+ptm score was used for further analyses.

**Supplementary Data 11: AlphaFold2 structure prediction of the complex of *Bacillus subtilis* AcsA and *B. subtilis* AcuA.** AlphaFold2 version 2.3.1 was used to predict the structure (Supplementary Fig. 2; Supplementary Fig.3). As output files, this PDB coordinate files was generated.

**Supplementary Data 12: Data quality and confidence score for the AlphaFold2 structure prediction complex of *Bacillus subtilis* AcsA and *B. subtilis* AcuA.** To judge the quality and confidence of the AlphaFold2 structure predictions for the multimers the iptm (interface ptm)+ptm (predicted TM)-scores were calculated. The model with the highest overall iptm+ptm score was used for further analyses.

**Supplementary Data 13: AlphaFold2 structure prediction of the complex of *Bacillus subtilis* AcsA K549Q and *B. subtilis* AcuA.** AlphaFold2 version 2.3.1 was used to predict the structure (Supplementary Fig. 2; Supplementary Fig.3). As output files, this PDB coordinate files was generated.

**Supplementary Data 14: Data quality and confidence score for the AlphaFold2 structure prediction complex of *Bacillus subtilis* AcsA K549Q and *B. subtilis* AcuA.** To judge the quality and confidence of the AlphaFold2 structure predictions for the multimers the iptm (interface ptm)+ptm (predicted TM)-scores were calculated. The model with the highest overall iptm+ptm score was used for further analyses.

**Supplementary Data 15: AlphaFold2 structure prediction of the complex of *Bacillus subtilis* AcsA K549R and *B. subtilis* AcuA.** AlphaFold2 version 2.3.1 was used to predict the structure (Supplementary Fig. 2; Supplementary Fig.3). As output files, this PDB coordinate files was generated.

**Supplementary Data 16: Data quality and confidence score for the AlphaFold2 structure prediction complex of *Bacillus subtilis* AcsA K549R and *B. subtilis* AcuA.** To judge the quality and confidence of the AlphaFold2 structure predictions for the multimers the iptm (interface ptm)+ptm (predicted TM)-scores were calculated. The model with the highest overall iptm+ptm score was used for further analyses.

**Supplementary Data 17: AlphaFold2 structure prediction of the complex of *Bacillus subtilis* AcsA E473A and *B. subtilis* AcuA.** AlphaFold2 version 2.3.1 was used to predict the structure (Supplementary Fig. 2; Supplementary Fig.3). As output files, this PDB coordinate files was generated.

**Supplementary Data 18: Data quality and confidence score for the AlphaFold2 structure prediction complex of *Bacillus subtilis* AcsA E473A and *B. subtilis* AcuA.** To judge the quality and confidence of the AlphaFold2 structure predictions for the multimers the iptm (interface ptm)+ptm (predicted TM)-scores were calculated. The model with the highest overall iptm+ptm score was used for further analyses.

**Supplementary Data 19: AlphaFold2 structure prediction of the complex of *Bacillus subtilis* AcsA V477A and *B. subtilis* AcuA.** AlphaFold2 version 2.3.1 was used to predict the structure (Supplementary Fig. 2; Supplementary Fig.3). As output files, this PDB coordinate files was generated.

**Supplementary Data 20: Data quality and confidence score for the AlphaFold2 structure prediction complex of *Bacillus subtilis* AcsA V477A and *B. subtilis* AcuA.** To judge the quality and confidence of the AlphaFold2 structure predictions for the multimers the iptm (interface ptm)+ptm (predicted TM)-scores were calculated. The model with the highest overall iptm+ptm score was used for further analyses.

**Supplementary Data 21: AlphaFold2 structure prediction of the complex of *Bacillus subtilis* AcsA and *B. subtilis* AcuA E85A.** AlphaFold2 version 2.3.1 was used to predict the structure (Supplementary Fig. 2; Supplementary Fig.3). As output files, this PDB coordinate files was generated.

**Supplementary Data 22: Data quality and confidence score for the AlphaFold2 structure prediction complex of *Bacillus subtilis* AcsA and *B. subtilis* AcuA E85A.** To judge the quality and confidence of the AlphaFold2 structure predictions for the multimers the iptm (interface ptm)+ptm (predicted TM)-scores were calculated. The model with the highest overall iptm+ptm score was used for further analyses.

**Supplementary Data 23: AlphaFold2 structure prediction of the complex of *Bacillus subtilis* AcsA and *B. subtilis* AcuA E85Q.** AlphaFold2 version 2.3.1 was used to predict the structure (Supplementary Fig. 2; Supplementary Fig.3). As output files, this PDB coordinate files was generated.

**Supplementary Data 24: Data quality and confidence score for the AlphaFold2 structure prediction complex of *Bacillus subtilis* AcsA and *B. subtilis* AcuA E85Q.** To judge the quality and confidence of the AlphaFold2 structure predictions for the multimers the iptm (interface ptm)+ptm (predicted TM)-scores were calculated. The model with the highest overall iptm+ptm score was used for further analyses.

**Supplementary Data 25: AlphaFold2 structure prediction of the complex of *Bacillus subtilis* AcsA and *B. subtilis* AcuA E97A.** AlphaFold2 version 2.3.1 was used to predict the structure (Supplementary Fig. 2; Supplementary Fig.3). As output files, this PDB coordinate files was generated.

**Supplementary Data 26: Data quality and confidence score for the AlphaFold2 structure prediction complex of *Bacillus subtilis* AcsA and *B. subtilis* AcuA E97A.** To judge the quality and confidence of the AlphaFold2 structure predictions for the multimers the iptm (interface ptm)+ptm (predicted TM)-scores were calculated. The model with the highest overall iptm+ptm score was used for further analyses.

**Supplementary Data 27: AlphaFold2 structure prediction of the complex of *Bacillus subtilis* AcsA and *B. subtilis* AcuA E97Q.** AlphaFold2 version 2.3.1 was used to predict the structure (Supplementary Fig. 2; Supplementary Fig.3). As output files, this PDB coordinate files was generated.

**Supplementary Data 28: Data quality and confidence score for the AlphaFold2 structure prediction complex of *Bacillus subtilis* AcsA and *B. subtilis* AcuA E97Q.** To judge the quality and confidence of the AlphaFold2 structure predictions for the multimers the iptm (interface ptm)+ptm (predicted TM)-scores were calculated. The model with the highest overall iptm+ptm score was used for further analyses.

**Supplementary Data 29: AlphaFold2 structure prediction of the complex of *Bacillus subtilis* AcsA and *B. subtilis* AcuA E85Q E97Q.** AlphaFold2 version 2.3.1 was used to predict the structure (Supplementary Fig. 2; Supplementary Fig.3). As output files, this PDB coordinate files was generated.

**Supplementary Data 30: Data quality and confidence score for the AlphaFold2 structure prediction complex of *Bacillus subtilis* AcsA and *B. subtilis* AcuA E85Q E97Q.** To judge the quality and confidence of the AlphaFold2 structure predictions for the multimers the iptm (interface ptm)+ptm (predicted TM)-scores were calculated. The model with the highest overall iptm+ptm score was used for further analyses.

**Supplementary Data 31: AlphaFold2 structure prediction of the complex of *Bacillus subtilis* AcsA and *B. subtilis* AcuA E102A.** AlphaFold2 version 2.3.1 was used to predict the structure (Supplementary Fig. 2; Supplementary Fig.3). As output files, this PDB coordinate files was generated.

**Supplementary Data 32: Data quality and confidence score for the AlphaFold2 structure prediction complex of *Bacillus subtilis* AcsA and *B. subtilis* AcuA E102A.** To judge the quality and confidence of the AlphaFold2 structure predictions for the multimers the iptm (interface ptm)+ptm (predicted TM)-scores were calculated. The model with the highest overall iptm+ptm score was used for further analyses.

**Supplementary Data 33: AlphaFold2 structure prediction of the complex of *Bacillus subtilis* AcsA and *B. subtilis* AcuA E102Q.** AlphaFold2 version 2.3.1 was used to predict the structure (Supplementary Fig. 2; Supplementary Fig.3). As output files, this PDB coordinate files was generated.

**Supplementary Data 34: Data quality and confidence score for the AlphaFold2 structure prediction complex of *Bacillus subtilis* AcsA and *B. subtilis* AcuA E102Q.** To judge the quality and confidence of the AlphaFold2 structure predictions for the multimers the iptm (interface ptm)+ptm (predicted TM)-scores were calculated. The model with the highest overall iptm+ptm score was used for further analyses.

**Supplementary Data 35: AlphaFold2 structure prediction of the complex of *Bacillus subtilis* AcsA and *B. subtilis* AcuA E135A.** AlphaFold2 version 2.3.1 was used to predict the structure (Supplementary Fig. 2; Supplementary Fig.3). As output files, this PDB coordinate files was generated.

**Supplementary Data 36: Data quality and confidence score for the AlphaFold2 structure prediction complex of *Bacillus subtilis* AcsA and *B. subtilis* AcuA E135A.** To judge the quality and confidence of the AlphaFold2 structure predictions for the multimers the iptm (interface ptm)+ptm (predicted TM)-scores were calculated. The model with the highest overall iptm+ptm score was used for further analyses.

**Supplementary Data 37: AlphaFold2 structure prediction of the complex of *Bacillus subtilis* AcsA and *B. subtilis* AcuA E135Q.** AlphaFold2 version 2.3.1 was used to predict the structure (Supplementary Fig. 2; Supplementary Fig.3). As output files, this PDB coordinate files was generated.

**Supplementary Data 38: Data quality and confidence score for the AlphaFold2 structure prediction complex of *Bacillus subtilis* AcsA and *B. subtilis* AcuA E135Q.** To judge the quality and confidence of the AlphaFold2 structure predictions for the multimers the iptm (interface ptm)+ptm (predicted TM)-scores were calculated. The model with the highest overall iptm+ptm score was used for further analyses.

**Supplementary Data 39: AlphaFold2 structure prediction of the complex of *Bacillus subtilis* AcsA and *B. subtilis* AcuA E97Q E125Q.** AlphaFold2 version 2.3.1 was used to predict the structure (Supplementary Fig. 2; Supplementary Fig.3). As output files, this PDB coordinate files was generated.

**Supplementary Data 40: Data quality and confidence score for the AlphaFold2 structure prediction complex of *Bacillus subtilis* AcsA and *B. subtilis* AcuA E97Q E135Q.** To judge the quality and confidence of the AlphaFold2 structure predictions for the multimers the iptm (interface ptm)+ptm (predicted TM)-scores were calculated. The model with the highest overall iptm+ptm score was used for further analyses.

**Supplementary Data 41: AlphaFold2 structure prediction of the complex of *Bacillus subtilis* AcsA and *B. subtilis* AcuA H139A.** AlphaFold2 version 2.3.1 was used to predict the structure (Supplementary Fig. 2; Supplementary Fig.3). As output files, this PDB coordinate files was generated.

**Supplementary Data 42: Data quality and confidence score for the AlphaFold2 structure prediction complex of *Bacillus subtilis* AcsA and *B. subtilis* AcuA H139A.** To judge the quality and confidence of the AlphaFold2 structure predictions for the multimers the iptm (interface ptm)+ptm (predicted TM)-scores were calculated. The model with the highest overall iptm+ptm score was used for further analyses.

**Supplementary Data 43: AlphaFold2 structure prediction of the complex of *Bacillus subtilis* AcsA and *B. subtilis* AcuA W140A.** AlphaFold2 version 2.3.1 was used to predict the structure (Supplementary Fig. 2; Supplementary Fig.3). As output files, this PDB coordinate files was generated.

**Supplementary Data 44: Data quality and confidence score for the AlphaFold2 structure prediction complex of *Bacillus subtilis* AcsA and *B. subtilis* AcuA W140A.** To judge the quality and confidence of the AlphaFold2 structure predictions for the multimers the iptm (interface ptm)+ptm (predicted TM)-scores were calculated. The model with the highest overall iptm+ptm score was used for further analyses.

**Supplementary Data 45: AlphaFold2 structure prediction of the complex of *Bacillus subtilis* AcsA and *B. subtilis* AcuA W140F.** AlphaFold2 version 2.3.1 was used to predict the structure (Supplementary Fig. 2; Supplementary Fig.3). As output files, this PDB coordinate files was generated.

**Supplementary Data 46: Data quality and confidence score for the AlphaFold2 structure prediction complex of *Bacillus subtilis* AcsA and *B. subtilis* AcuA W140F.** To judge the quality and confidence of the AlphaFold2 structure predictions for the multimers the iptm (interface ptm)+ptm (predicted TM)-scores were calculated. The model with the highest overall iptm+ptm score was used for further analyses.
